# Murine Gamma-Herpesvirus 68 Hijacks MAVS and IKKβ to Initiate Lytic Replication

**DOI:** 10.1371/journal.ppat.1001001

**Published:** 2010-07-29

**Authors:** Xiaonan Dong, Hao Feng, Qinmiao Sun, Haiyan Li, Ting-Ting Wu, Ren Sun, Scott A. Tibbetts, Zhijian J. Chen, Pinghui Feng

**Affiliations:** 1 Department of Microbiology, UT Southwestern Medical Center, Dallas, Texas, United States of America; 2 The State Key Laboratory of Biomembrane and Membrane Biotechnology, Institute of Zoology, Chinese Academy of Sciences, Chao Yang District, Beijing, People's Republic of China; 3 Department of Microbiology and Immunology, Louisiana State University Health Science Center, Shreveport, Louisiana, United States of America; 4 Department of Molecular and Medical Pharmacology, University of California Los Angeles, Los Angeles, California, United States of America; 5 Department of Molecular Biology, UT Southwestern Medical Center, Dallas, Texas, United States of America; Oregon Health & Science University, United States of America

## Abstract

Upon viral infection, the mitochondrial antiviral signaling (MAVS)-IKKβ pathway is activated to restrict viral replication. Manipulation of immune signaling events by pathogens has been an outstanding theme of host-pathogen interaction. Here we report that the loss of MAVS or IKKβ impaired the lytic replication of gamma-herpesvirus 68 (γHV68), a model herpesvirus for human Kaposi's sarcoma-associated herpesvirus and Epstein-Barr virus. γHV68 infection activated IKKβ in a MAVS-dependent manner; however, IKKβ phosphorylated and promoted the transcriptional activation of the γHV68 replication and transcription activator (RTA). Mutational analyses identified IKKβ phosphorylation sites, through which RTA-mediated transcription was increased by IKKβ, within the transactivation domain of RTA. Moreover, the lytic replication of recombinant γHV68 carrying mutations within the IKKβ phosphorylation sites was greatly impaired. These findings support the conclusion that γHV68 hijacks the antiviral MAVS-IKKβ pathway to promote viral transcription and lytic infection, representing an example whereby viral replication is coupled to host immune activation.

## Author Summary

Innate immunity represents the first line of defense against pathogen infection. Recent studies uncovered an array of sensors that detect pathogen-associated molecular patterns and induce antiviral cytokine production via two closely related kinase complexes, i.e., the IKKα/β/γ and TBK-1/IKKε. To counteract host immune defense, herpesviruses have evolved diverse strategies to evade, manipulate, and exploit host immune responses. Here we report that infection by murine gamma-herpesvirus 68 (γHV68), a model gamma-herpesvirus for human Kaposi's sarcoma-associated herpesvirus and Epstein-Barr virus, activated the IKKβ kinase and IKKβ was usurped to promote viral transcriptional activation. As such, uncoupling IKKβ from transcriptional activation by biochemical and genetic approaches impaired γHV68 lytic replication. Our study represents an example whereby viral lytic replication is coupled to host innate immune activation and sheds light on herpesvirus exploitation of immune responses.

## Introduction

Host cells activate innate immune signaling pathways to defend against invading pathogens. Pattern recognition receptors, including Toll-like receptors and cytosolic sensors (such as NOD-like receptors and RIG-I-like receptors), recognize pathogen-associated structural components and initiate signal transduction that leads to the biosynthesis and secretion of pro-inflammatory cytokines and interferons, thereby mounting a potent host immune response [1], [2]. To survive within an infected host, viruses have evolved intricate strategies to counteract host immune responses. Herpesviruses and poxviruses have large genomes and therefore have the capacity to encode numerous proteins that modulate host immune responses.

**M**itochonrial **a**nti**v**iral **s**ignaling (MAVS, also known as IPS-1, VISA, and CARDIF) protein serves as an adaptor to activate both the NFκB and interferon regulatory factor (IRF) pathways [3], [4], [5], [6]. MAVS relays signals from RIG-I and MDA-5, cytosolic sensors that recognize viral dsRNA or ssRNA bearing 5′-triphosphate [7], [8], to the IKKα/β/γ and TBK-1/IKKε (also known as IKKi) kinase complexes [4], [6]. IKKα/β, together with the scaffold protein IKKγ, phosphorylates the inhibitor of NFκB (IκB) and promotes its subsequent ubiquitination and degradation by the proteasome, thereby unleashing NFκB that translocates into the nucleus to activate gene expression of pro-inflammatory cytokines [9], [10]. By contrast, TBK-1 and IKKε directly phosphorylate a serine/threonine-rich sequence within the carboxyl termini of IRF3 and IRF7, leading to the dimerization and nuclear translocation of these transcription factors [11], [12]. Together with NFκB and c-Jun/ATF-2, IRF3 and IRF7 bind to the interferon (IFN)-β enhancer and initiate the transcription of IFN-β [13], [14]. Ultimately, these signaling events promote cytokine and interferon production, establishing an antiviral state in infected cells. Although it is not clear how MAVS activates these immune kinases, recent findings have established the vital roles of MAVS in host antiviral innate immunity [15]. Interestingly, the mitochondrial localization of MAVS is critical for its ability to activate downstream signaling events. As such, various RNA viruses, exemplified by human hepatitis C virus (HCV), encode proteases that cleave MAVS from the outer membrane of the mitochondrion, thereby disarming MAVS-dependent signaling cascades and the host antiviral innate immunity [6], [16], [17], [18].

Murine gamma-herpesvirus 68 (γHV68 or MHV-68) is closely related to human Kaposi's sarcoma-associated herpesvirus (KSHV) and Epstein-Barr virus (EBV) [19]. KSHV and EBV are lymphotropic DNA viruses that are causally linked to malignancies of lymphoid or endothelial/epithelial origin, including lymphoma, nasopharyngeal carcinoma, and Kaposi's sarcoma [20], [21]. Persisting within host immune cells, KSHV and EBV are known to evade, manipulate, and exploit host immune pathways [22], [23]. Emerging studies suggest that γ-herpesviruses may usurp host innate immune responses for their infection [24], [25], [26]. However, it is not known how human KSHV and EBV manipulate innate immune pathways *in vivo*. Such investigations are greatly hampered by the lack of permissive cell lines and animal models for both KSHV and EBV. By contrast, γHV68 infection in laboratory mice leads to a robust acute infection in the lung and a long-term latent infection in the spleen. For murine γHV68 and human KSHV, the **r**eplication and **t**ranscription **a**ctivator (RTA, encoded by ORF50) is necessary and sufficient to initiate lytic replication from latently-infected cells, supporting the notion that RTA integrates diverse signaling pathways to initiate lytic replication [27], [28], [29]. Using γHV68 as a surrogate for human KSHV and EBV, we have unexpectedly discovered that γHV68 activated IKKβ to phosphorylate RTA and promote RTA transcriptional activation, thereby increasing viral gene transcription and lytic replication. As such, RTA phosphorylation by IKKβ couples γHV68 gene expression and lytic replication to host innate immune activation, representing the first example whereby a virus hijacks the antiviral MAVS-IKKβ pathway to promote its lytic replication.

## Results

### MAVS is Necessary for Efficient γHV68 Lytic Replication

**Figure 1:**
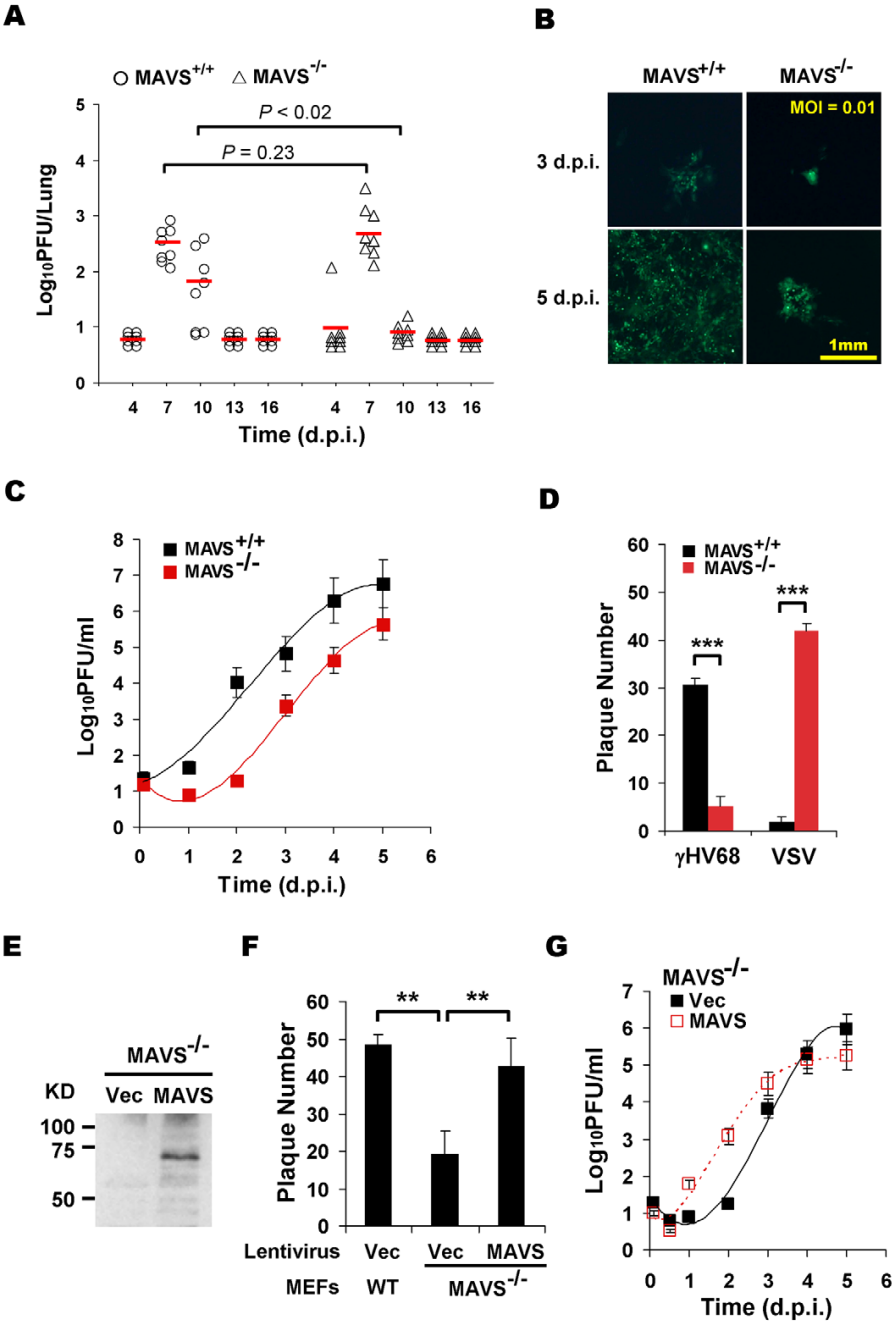
MAVS deficiency reduces γHV68 lytic replication. MAVS wild-type (MAVS^+/+^) or knockout (MAVS^−/−^) mice were intranasally (i.n.) infected with 40 PFU γHV68. The lungs were harvested at 4, 7, 10, 13, and 16 days post-infection (d.p.i.) and viral titer was determined by a plaque assay. (B) Mouse embryonic fibroblasts (MEFs) were infected with a GFP-marked γHV68 K3/GFP at multiplicities of infection (MOI) of 0.01. Viral replication in MAVS^+/+^ and MAVS^−/−^ MEFs were photographed. (C) MEFs were infected with γHV68 K3/GFP as in (B) and viral titers were determined by a plaque assay. Data represent three independent experiments and error bars denote standard error of the mean (SEM). (D) MAVS^+/+^ and MAVS^−/−^ MEFs were infected with 120 PFU γHV68 K3/GFP or 5 PFU vesicular stomatitis virus (VSV), and plaques were counted. Data represent the mean ± SEM of three independent experiments. (E to G) MAVS^−/−^ MEFs were respectively infected with control lentivirus (Vec) or lentivirus containing the Flag-tagged human MAVS (MAVS), and selected with puromycin. (E) MAVS expression was confirmed by immunoprecipitation and immunoblot with anti-Flag antibody. (F) γHV68 plaque assays were performed as in (D). (G) Reconstituted MAVS^−/−^ MEFs as indicated were infected with γHV68 K3/GFP (MOI = 0.01), and viral multi-step growth was determined by a plaque assay. Statistical significance in (A), (D), and (F): *, *P*<0.05; **, *P*<0.02; ***, *P*<0.005.

To investigate the roles of MAVS in γHV68 infection, wild-type (MAVS^+/+^), heterozygous (MAVS^+/−^), and knockout (MAVS^−/−^) mice were intranasally (i.n.) infected with 40 plaque-forming unit (PFU) γHV68. γHV68 acute infection in the lung was measured by plaque assays at 4, 7, 10, 13, and 16 days post-infection (d.p.i.). In MAVS^+/+^ mice, γHV68 titers peaked at 7 d.p.i. with approximately 500 PFU/lung and declined to 100 PFU/lung at 10 d.p.i. Viral load was undetectable by 13 d.p.i., indicating that γHV68 acute infection in the lung had been cleared (Figure 1A). Similar viral loads in the lungs of heterozygous mice (MAVS^+/−^) were observed (data not shown). Surprisingly, although viral loads at 7 d.p.i. in the lungs of MAVS^−/−^ mice were comparable to those in the lungs of MAVS^+/+^ mice, γHV68 was nearly undetectable at 10 d.p.i. (Figure 1A). By contrast, γHV68 latent infection as characterized by viral genome frequency, persistent lytic replication, and reactivation was similar in splenocytes of MAVS^+/+^ and MAVS^−/−^ mice at 16 and 45 d.p.i. (Figure S1). These observations suggest that MAVS plays a specific role(s) in γHV68 acute infection.

To determine whether γHV68 infection altered MAVS expression, we infected BL/6 mice intranasally with a high dose (1×10^5^ PFU) of γHV68, presumably permitting synchronized and maximal infection of lung epithelial cells. MAVS mRNA levels were determined by quantitative real-time PCR (qRT-PCR). The levels of MAVS mRNA were transiently increased at 2.5 and 5 d.p.i. in the lung and spleen, respectively (Figure S2A). Interestingly, the up-regulation of MAVS mRNA preceded that of viral RTA mRNA (Figure S2A and S2B), and that higher viral RTA mRNA levels tightly correlated with higher MAVS mRNA levels at 2.5 and 5 d.p.i., when MAVS mRNA levels peaked in the lung and spleen (Figure S2C). Together with the reduced viral load in the lungs of MAVS^−/−^ mice (Figure 1A), these results suggest that MAVS is necessary for efficient lytic replication in mice and that the transiently induced MAVS expression by γHV68 infection may facilitate viral lytic replication *in vivo*.

To investigate the roles of MAVS in γHV68 infection, we then assessed the effects of MAVS-deficiency on γHV68 lytic replication *ex vivo*. Mouse embryonic fibroblasts (MEFs) were infected with a GFP-marked recombinant γHV68 (γHV68 K3/GFP) and viral replication was examined by fluorescence microscopy and plaque assays. Surprisingly, γHV68 displayed delayed replication kinetics in MAVS^−/−^ MEFs compared to MAVS^+/+^ MEFs at multiplicities of infection (MOI) of 0.01 and 0.1 (Figure 1B, 1C and S3). To quantitatively determine the effect of MAVS on γHV68 lytic infection, we examined γHV68 lytic replication in MAVS^+/+^ and MAVS^−/−^ MEFs by plaque assays. In fact, γHV68 formed approximately four-fold more plaques in MAVS^+/+^ MEFs than those in MAVS^−/−^ MEFs, indicative of reduced initiation of lytic replication in MAVS-deficient MEFs (Figure 1D, S4A, and S4B). Interestingly, the plaque size of γHV68 was equivalent in MAVS^+/+^ and MAVS^−/−^ MEFs (Figure S4C and S4D). To test whether MAVS^−/−^ MEFs are defective in supporting viral lytic replication in general, we examined the lytic replication of vesicular stomatitis virus (VSV), a prototype RNA virus, with a plaque assay. Consistent with an antiviral activity of MAVS against RNA viruses, VSV formed 10-fold more plaques in MAVS^−/−^ MEFs than those in MAVS^+/+^ MEFs (Figure 1D). The diminished lytic replication of γHV68 in MAVS-deficient MEFs is consistent with the reduced acute infection observed in the lung. To test whether exogenously expressed MAVS is able to restore γHV68 lytic replication, we generated lentivirus in 293T cells and MEFs stably expressing human MAVS (hMAVS) was established with puromycin selection (Figure 1E). As shown in Figure 1F and 1G, exogenous hMAVS restored γHV68 lytic replication by a plaque assay and multi-step growth curves. Nevertheless, these results together support the conclusion that MAVS is necessary for efficient γHV68 lytic replication *in vivo* and *ex vivo*.

### The IKKβ/γ Complex is an Effector Downstream of MAVS in γHV68 Lytic Replication

**Figure 2:**
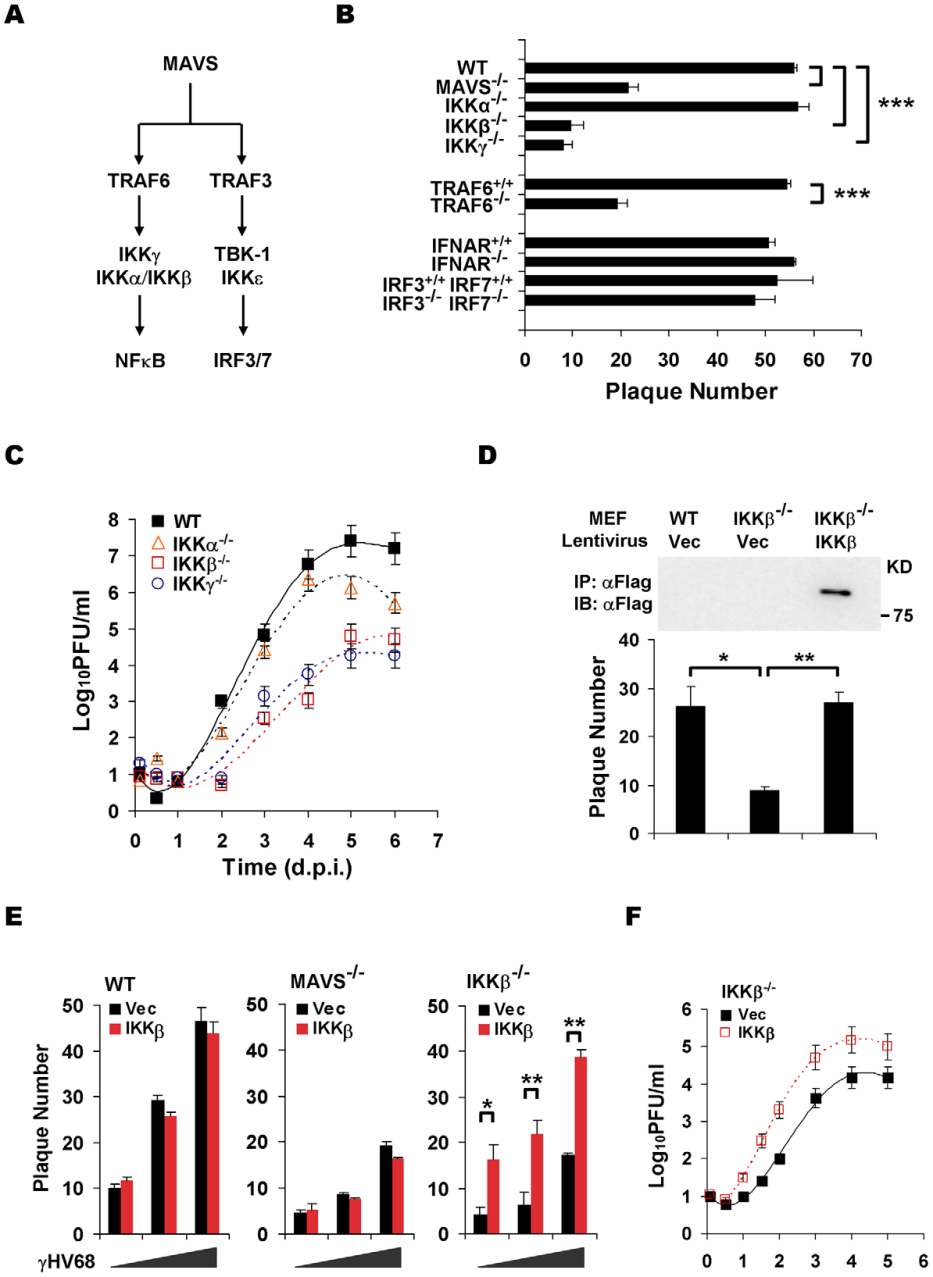
The MAVS-IKKβ pathway is necessary for efficient γHV68 lytic replication *ex vivo*. (A) Two known pathways, the IKKα/β/γ-NFκB and TBK-1/IKKε-IRF pathways, downstream of MAVS. (B) The initiation of γHV68 lytic replication in wild-type (WT) MEFs and MAVS^−/−^, IKKα^−/−^, IKKβ^−/−^, IKKγ^−/−^, TRAF6^−/−^, IFNAR^−/−^, and IRF3^−/−^IRF7^−/−^ (double knockout) MEFs was assessed by a plaque assay. Data represent the mean ± SEM of three independent experiments. (C) Multi-step growth properties of γHV68 (MOI = 0.01) in wild-type MEFs and IKKβ^−/−^, IKKγ^−/−^, and IKKα^−/−^ MEFs were examined by plaque assays. Data represents three independent experiments. (D to F) Wild-type, MAVS^−/−^, and IKKβ^−/−^ MEFs were respectively infected with control lentivirus (Vec) or lentivirus containing the Flag-tagged IKKβ (IKKβ), and selected with puromycin. (D) IKKβ expression was confirmed by immunoprecipitation and immunoblot with anti-Flag antibody (top). γHV68 plaque assays were performed as in (B). (E) Reconstituted MEFs of indicated genotypes were used for γHV68 plaque assays as in (B) with increasing doses of γHV68. Data represent the mean ± SEM of three independent experiments. (F) Reconstituted IKKβ^−/−^ MEFs as indicated were infected with γHV68 K3/GFP (MOI = 0.01), and viral multi-step growth was determined by a plaque assay. Statistical significance (*P*-value) in (B), (D), and (E) was calculated with two-tailed unpaired Student's *t*-test: *, *P*<0.05; **, *P*<0.02; ***, *P*<0.005.

Two known pathways, the IKKα/β/γ-NFκB and TBK-1/IKKε-IRF3/7 pathway, have been characterized downstream of MAVS (Figure 2A) [3], [4]. We therefore used MEFs deficient in key components of aforementioned pathways to identify downstream effectors of MAVS that are critical for γHV68 lytic infection. Plaque assays and multi-step growth curves of γHV68 lytic infection showed that deficiency in TRAF6, IKKγ, and IKKβ, but not deficiency in the closely related IKKα, recapitulated phenotypes of MAVS deficiency (Figure 2B and 2C). Notably, TRAF6 is necessary for MAVS to activate IKKβ that requires IKKγ, a scaffold protein for both IKKα and IKKβ [5]. By contrast, deficiency in type I IFN receptor (IFNAR) and double deficiency in IRF3 and IRF7 had no discernable effect on the plaque numbers of γHV68 in MEFs, indicating that the IRF-IFN signaling pathway is not critical for the initiation of γHV68 lytic replication (Figure 2B). Furthermore, the exogenous IKKβ expression reconstituted by lentivirus restored the lytic replication of γHV68 as determined by a plaque assay and multi-step growth curves (Figure 2D, 2E, and 2F). Interestingly, the expression of IKKβ in MAVS^−/−^ did not increase γHV68 lytic replication by a plaque assay (Figure 2E), suggesting that the MAVS-dependent activation of IKKβ, rather than the absolute expression level of IKKβ, is crucial for efficient γHV68 lytic replication. Additionally, exogenous IKKβ did not increase γHV68 plaque numbers in MAVS^+/+^ MEFs (Figure 2E), implying that endogenous IKKβ is sufficient to support efficient γHV68 lytic replication. Of note, lentivirus infection reduces the difference of γHV68 plaque forming capacity in wild-type MEFs and in MEFs deficient in MAVS and IKKβ (Figure 1F and 2D). Collectively, these data indicate that the MAVS-dependent IKKβ activation is critical for efficient γHV68 lytic replication.

**Figure 3:**
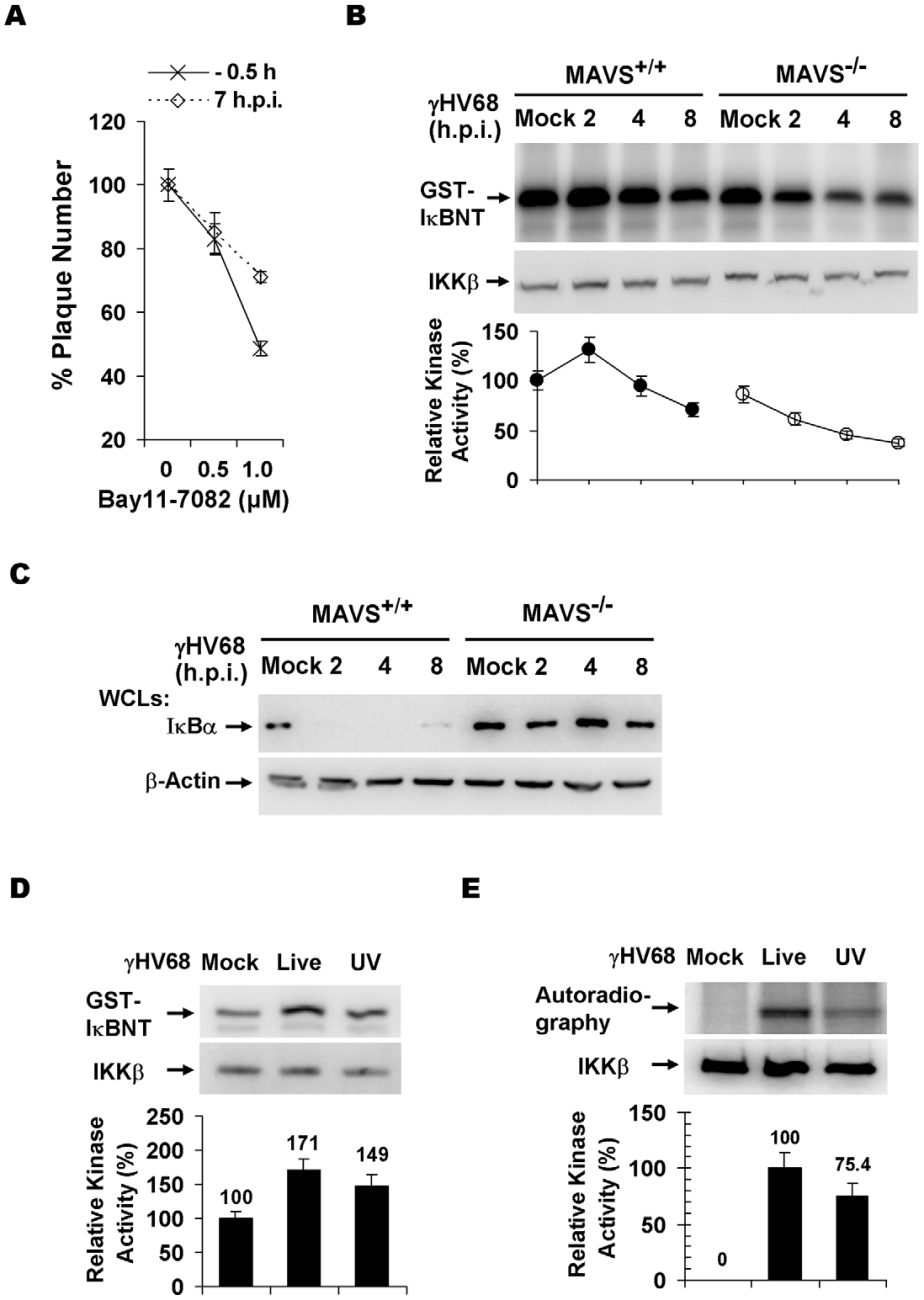
γHV68 infection activates IKKβ in a MAVS-dependent manner. (A) Wild-type MEFs were treated with the IKKβ inhibitor, Bay11-7082, for 30 min at 0.5 h before infection or 7 h post-infection (h.p.i.) with γHV68. Cells were washed with medium and incubated for plaque formation. Plaques formed at 6 d.p.i. were counted. Data represent the mean ± SEM. (B) MEFs were infected with γHV68 (MOI = 10) and whole cell lysates of MEFs at indicated time points after γHV68 infection were precipitated with anti-IKKβ antibody. One half of IKKβ was used for an *in vitro* kinase assay with GST-IκBNT (amino terminal 50 amino acids of IκBα) (top) or analyzed by immunoblot (middle). Relative intensity of phosphorylated GST-IκBNT was normalized to IKKβ protein (bottom). (C) γHV68 infection was carried out as in (B) and whole cell lysates were analyzed by immunoblot with anti-IκBα (top) and β-actin (bottom). (D and E) Equal amount of live (MOI = 10) or UV-inactivated (UV) γHV68 was used to infect wild-type MEFs. The IKKβ kinase activity was assessed as in (B) and whole cell lysates were analyzed by immunoblot as in (C) for IκBα and β-actin. Graphs at the bottom show normalized IKKβ kinase activity (D) and IκBα protein (E).

To assess whether the kinase activity of IKKβ is important for γHV68 lytic infection, we performed plaque assays with or without the specific IKKβ inhibitor, Bay11-7082 (Bay11). This experiment revealed that Bay11 reduced the plaque number of γHV68 in a dose-dependent manner (Figure 3A). Whereas treatment with 1 µM of Bay11 at 0.5 h before infection reduced γHV68 plaque number by 52%, the same treatment at 7 h post-infection (h.p.i.) reduced the plaque number by 29%, emphasizing the important roles of IKKβ during early γHV68 infection (Figure 3A). We further examined IKKβ activity by an *in vitro* kinase assay with IKKβ precipitated from MAVS^+/+^ and MAVS^−/−^ MEFs infected with γHV68. The IKKβ kinase activity was transiently and moderately increased in MAVS^+/+^ MEFs, however, it was drastically diminished in MAVS^−/−^ MEFs after γHV68 infection (Figure 3B). The activation of IKKβ was further supported by the rapid degradation of IκBα concurrent to IKKβ activation by γHV68 infection in MAVS^+/+^ MEFs, but not in MAVS^−/−^ MEFs (Figure 3C). To test whether UV-inactivated virus is able to trigger IKKβ activation, we examined the levels of IKKβ kinase activity and IκBα in MAVS^+/+^ MEFs by *in vitro* kinase and immunoblot assays, respectively. Interestingly, UV-inactivated γHV68 activated IKKβ and reduced IκBα protein levels, although less efficiently than live γHV68 (Figure 3D and 3E). This observation suggests that γHV68 lytic replication is necessary to activate the MAVS-IKKβ pathway. Alternatively, UV treatment may damage or disrupt viral structural components whose integrity is necessary to activate the MAVS-IKKβ pathway.

MAVS activation by RNA viruses is known to increase the expression of pro-inflammatory cytokines and interferons. However, γHV68 appears to be a poor inducer for these antiviral molecules, suggesting that γHV68 evades signaling events downstream of the MAVS adaptor. Indeed, γHV68 infection failed to up-regulate the expression of IFN-β (Figure S5A). In agreement with this observation, γHV68 RTA, similar to KSHV RTA [30], is sufficient to reduce IRF3 expression (Figure S5B). Meanwhile, it was previously shown that γHV68 infection did not significantly activate NFκB during early infection [31], suggesting that γHV68 uncouples NFκB activation from activated IKKβ. Taken together, these results support the conclusion that γHV68 infection selectively activates IKKβ to promote viral lytic replication.

### The MAVS-IKKβ Pathway is Implicated in γHV68 Transcriptional Activation

**Figure 4:**
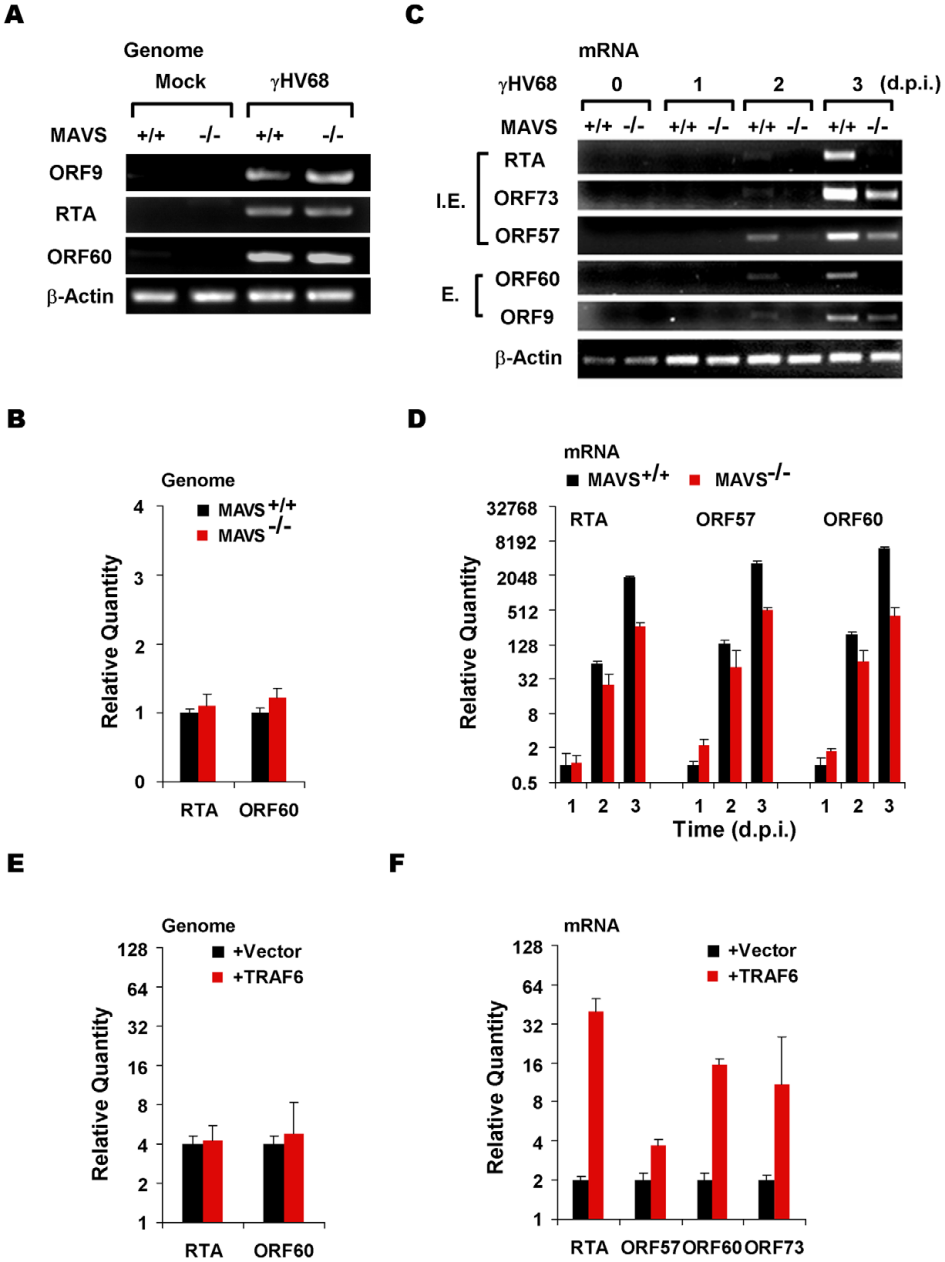
The MAVS-IKKβ pathway is important for γHV68 mRNA production. (A and B) MAVS^+/+^ and MAVS^−/−^ MEFs were infected with γHV68 (MOI = 0.01) and cells were harvested at two hours post-infection. Total DNA was extracted and viral genomes were analyzed by PCR and agarose gel electrophoresis (A) or quantitative real-time PCR (qRT-PCR) (B). (C and D) MEFs were infected with γHV68 as in (A). Total RNA was extracted and levels of γHV68 mRNA transcripts were examined by reverse transcription and PCR (C) or qRT-PCR (D) using primers specific for viral genes as indicated. I.E., immediate early; E, early. (E and F) 293T cells were transfected with the γHV68 BAC and a plasmid containing TRAF6. At 28 h post-transfection, levels of the γHV68 genome were determined by qRT-PCR (E) and levels of various γHV68 gene transcripts as indicated were determined by reverse transcription and qRT-PCR analyses (F). Data represent the mean ± SEM.

To discern the molecular mechanisms underlying the requirement of the MAVS-IKKβ pathway in γHV68 lytic infection, levels of γHV68 genomic DNA and mRNA were assessed by PCR or reverse transcription followed by real-time PCR analyses, respectively. At a low MOI (0.01), analyses by PCR (Figure 4A) and real-time PCR (Figure 4B) revealed comparable levels of viral genomes in MAVS^+/+^ and MAVS^−/−^ MEFs early after *de novo* infection, suggesting comparable viral entry into MAVS^+/+^ and MAVS^−/−^ MEFs. Interestingly, levels of viral mRNA transcripts representing immediate early (RTA, ORF73, and ORF57) and early (ORF60 and ORF9) gene products in MAVS^+/+^ MEFs were higher than those in MAVS^−/−^ MEFs as determined by reverse-transcriptase PCR (Figure 4C). Real-time PCR analyses with cDNA showed approximately 4- to 16-fold higher levels of γHV68 mRNA transcripts in MAVS^+/+^ MEFs compared to those in MAVS^−/−^ MEFs at 2 and 3 d.p.i. (Figure 4D). It has been shown that TRAF6 is necessary for MAVS to activate IKKβ [5] and exogenous TRAF6 is sufficient to activate IKKβ. To further examine the effects of the MAVS-IKKβ pathway on levels of γHV68 mRNA transcripts, a bacterial artificial chromosome (BAC) containing the γHV68 genome and a plasmid expressing TRAF6 were transfected into 293T cells. The effects of exogenous TRAF6 (that activates IKKβ) on viral transcription were determined by reverse transcription and real-time PCR. At 28 h post-transfection, a time point when immediate early and early genes are transcribed, exogenous TRAF6 efficiently increased the mRNA levels of γHV68 RTA, ORF57, ORF60, and ORF73, without discernable effect on levels of viral genomic DNA (Figure 4E and 4F). These results, obtained under conditions of loss of function (MAVS^−/−^ MEFs) and gain of function (TRAF6 expression), indicate that the activated IKKβ increases the levels of γHV68 mRNA transcripts.

### IKKβ Phosphorylates γHV68 RTA and Promotes γHV68 Transcription

**Figure 5:**
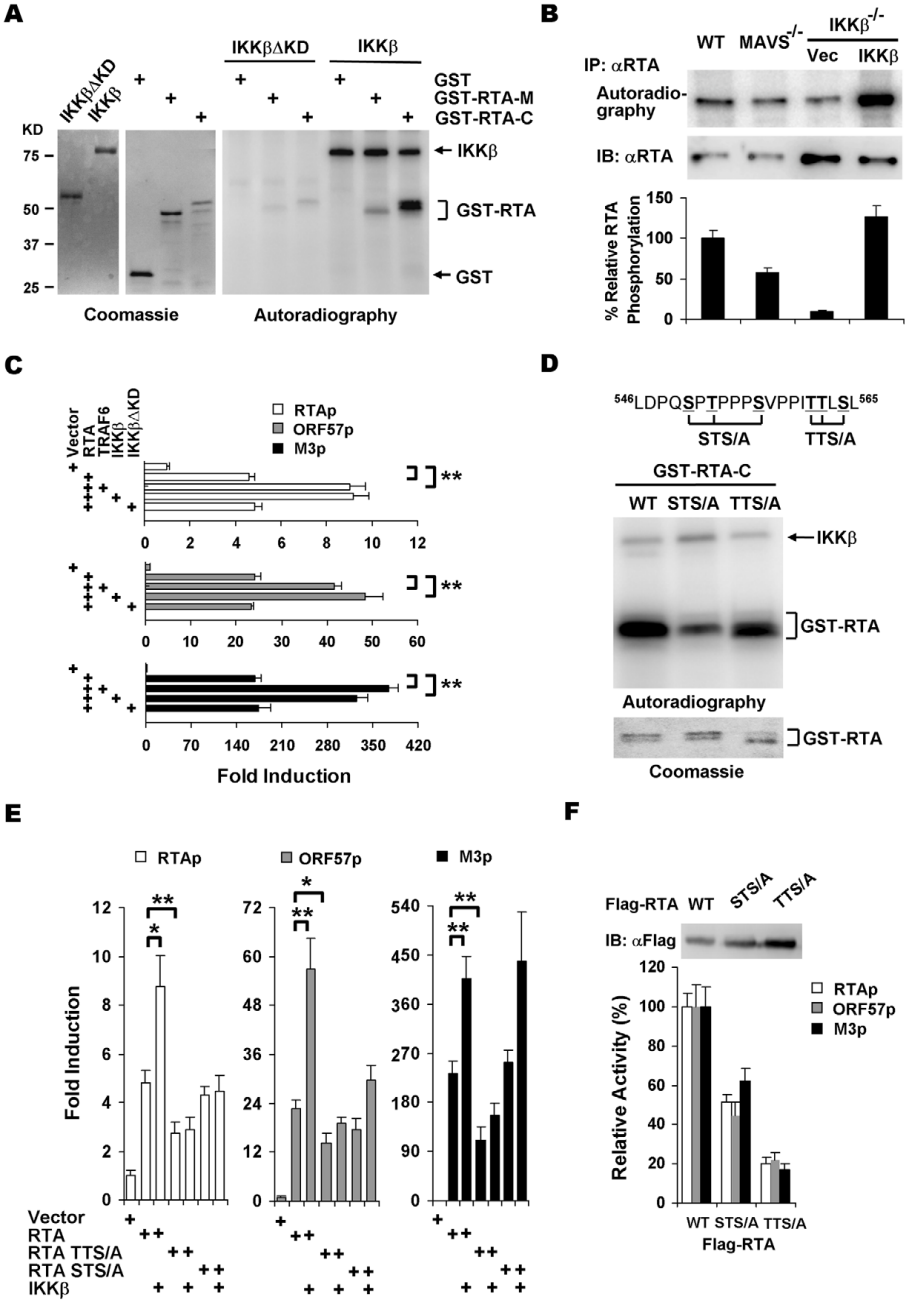
IKKβ phosphorylates and potentiates the transcription activity of γHV68 RTA. (A) IKKβ or IKKβΔKD purified from 293T cells (left) were incubated with [^32^P]γATP and bacterial GST fusion proteins containing RTA fragments (middle), and examined by autoradiography (right). (B) MEFs of indicated genotype were infected with γHV68 (MOI = 2) for 4 h, labeled with [^32^P]-orthophosphoric acid for 8 h. Whole cell lysates were precipitated with anti-RTA antibody and analyzed by autoradiography (top) or immunoblot with anti-RTA antibody (bottom). (C) 293T cells were transfected with reporter plasmids and plasmids containing RTA, TRAF6, IKKβ, and IKKβΔKD. Luciferase activity normalized against β-galactosidase activity was shown. (D) Phosphorylation of GST fusion proteins, containing the C-terminal 127 amino acids of wild-type RTA, STS/A, or TTS/A variants, by IKKβ was analyzed similarly as in (A). (E) 293T transfection and luciferase reporter assays were carried out as in (C). (F) Whole cell lysates of 293T transfected with plasmids containing wild-type RTA, STS/A, or TTS/A variants were analyzed by immunoblot (top) and used to normalize the basal transcriptional activity of wild-type RTA, the STS/A and TTS/A variants (bottom). Data in (C), (E), and (F) represent the mean ± SEM with indicated *P* values (*, *P*<0.05; **, *P*<0.02) of at least three independent experiments.

MAVS is an adaptor that activates IKKβ and the MAVS-dependent IKKβ increases γHV68 mRNA levels. We thus postulated that MAVS influences γHV68 transcription via its downstream IKKβ on RTA, because RTA, the master transcription activator, is critical for γHV68 lytic replication. To test this hypothesis, we examined whether IKKβ phosphorylates γHV68 RTA. IKKβ was purified from 293T cells and bacterial GST fusion proteins containing the RTA internal region (RTA-M, aa 335–466) or the RTA C-terminal transactivation domain (RTA-C, aa 457–583) were purified from *E.coli* (Figure 5A). In the presence of [^32^P]γATP, IKKβ efficiently transferred the phosphate group to GST-RTA-C. By contrast, GST was not phosphorylated and GST-RTA-M was weakly phosphorylated by IKKβ. Furthermore, the kinase domain deletion variant of IKKβ (IKKβΔKD) failed to phosphorylate GST-RTA-C and GST-RTA-M (Figure 5A), and IKKα had only residual kinase activity toward RTA-C (Figure S6). To confirm the MAVS- and IKKβ-dependent phosphorylation of RTA, RTA phosphorylation in γHV68-infected cells was analyzed by autoradiography and immunoblot. We found that MAVS- and IKKβ deficiency reduced RTA phosphorylation by 50% and 85%, respectively, while reconstituted IKKβ expression restored RTA phosphorylation to that of RTA in MAVS^+/+^ MEFs (Figure 5B). To assess the roles of phosphorylation of RTA in transcription regulation, luciferase reporter assays were carried out with plasmids containing RTA-responsive promoters of RTA, ORF57, and M3. As shown in Figure 5C, the transcription activity of RTA on all three promoters was significantly increased by exogenous TRAF6 and IKKβ, but not by the kinase dead variant IKKβΔKD, supporting the notion that IKKβ promotes RTA transcription activation via phosphorylation. When expressed to similar levels of IKKβ, IKKβΔKD had no significant effect on RTA transcriptional activation (Figure S7). Given that RTA is a substrate for IKKβ, we sought to examine whether RTA can physically associate with the IKKα/β/γ complex. However, we were unable to detect interaction between RTA and any of the three subunits of IKKα/β/γ by co-immunoprecipitation (data not shown), suggesting that the RTA interaction with the IKKα/β/γ complex is transient or mediated via additional cellular proteins.

To identify IKKβ phosphorylation sites, series of truncations from the C-terminus of RTA were constructed and purified as GST fusion proteins for *in vitro* kinase assays with IKKβ. These experiments demonstrated that the IKKβ phosphorylation sites were located within the region containing residues 540 through 567 (Figure S8). Given that IKKβ is a serine/threonine kinase, clusters of various serine/threonine residues were changed to alanines and RTA phosphorylation was assessed similarly. Two clusters of mutations, replacement of S_550_T_552_S_556_ (STS/A) and T_561_T_562_S_564_ (TTS/A) by alanines, reduced the phosphorylation levels of RTA-C by approximately 72% and 45%, respectively (Figure 5D and S8). These results indicate that the STS and TTS sequences represent two major IKKβ phosphorylation sites within the transactivation domain of RTA.

To further examine the roles of IKKβ phosphorylation in regulating RTA transcription activity, reporter assays with plasmids containing wild-type RTA, the STS/A and TTS/A variants were carried out with exogenously expressed IKKβ. The STS/A and TTS/A variants had lower basal activity to activate promoters of RTA, ORF57, and M3. Moreover, exogenous IKKβ failed to further stimulate the transcription activities of the STS/A and TTS/A variants to activate promoters of RTA and ORF57 (Figure 5E). Interestingly, the STS/A variant activated M3 promoter to the level of wild-type RTA with or without IKKβ, indicating that the STS site is dispensable for IKKβ to promote RTA transcriptional activity on the M3 promoter (Figure 5E). It is noteworthy that the STS/A and TTS/A variants were expressed at higher levels than wild-type RTA, the transcription activities of the STS/A and TTS/A variants were approximately 50% and 20% of that of wild-type RTA, respectively, when luciferase activity was normalized against protein levels (Figure 5F). Collectively, these results demonstrated that IKKβ promotes RTA transcriptional activation via phosphorylation of the TTS and STS sites within the transactivation domain.

### Impaired Lytic Replication of Recombinant γHV68 Carrying Mutations within the IKKβ Phosphorylation Sites

**Figure 6:**
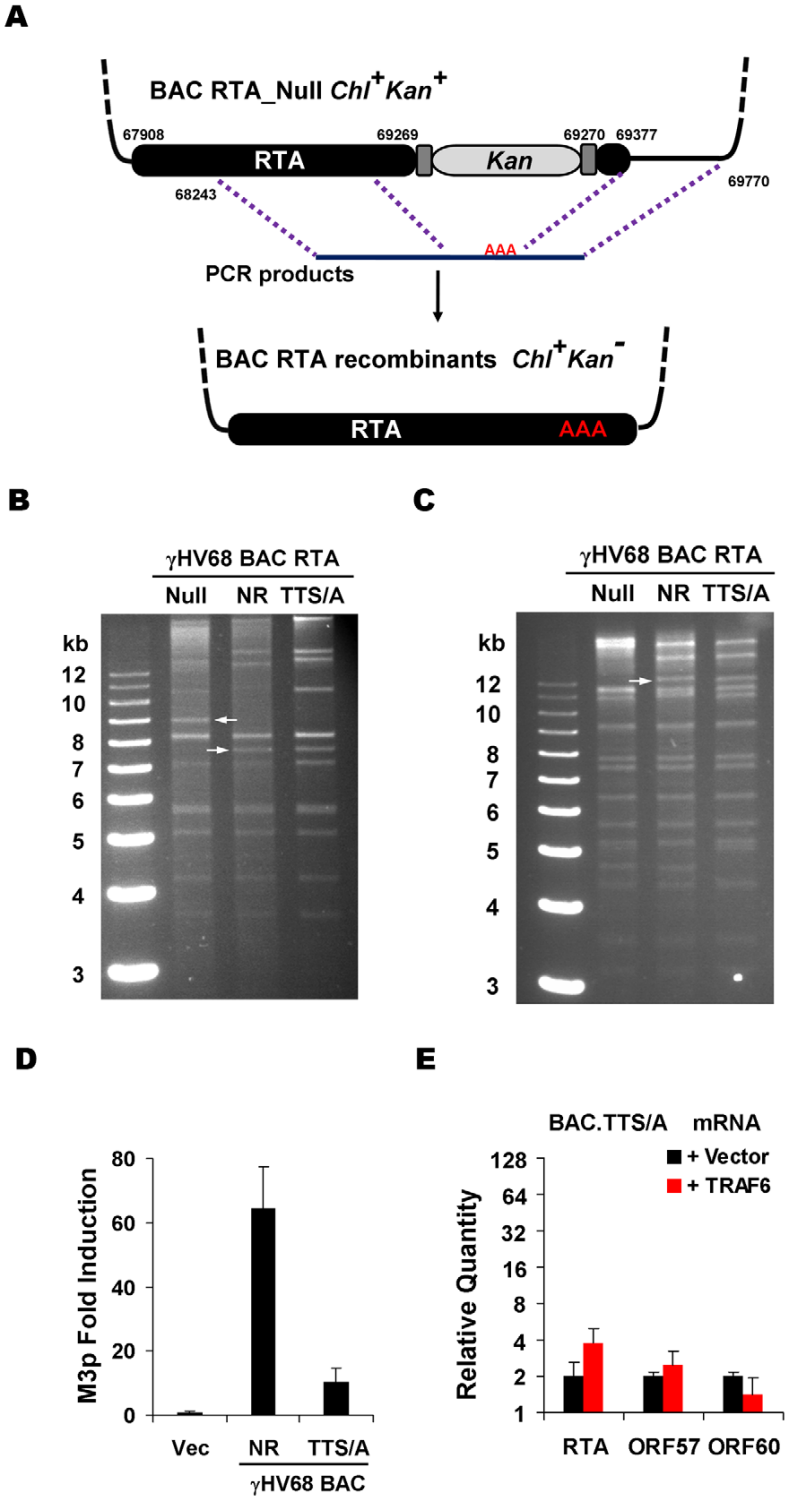
Generation and characterization of γHV68 BAC carrying the TTS/A mutation. (A) Diagram of the strategy to generate recombinant γHV68. Briefly, wild-type RTA or the STS/A and TTS/A alleles were PCR amplified with overlapping PCR primers. Purified PCR products were transfected into NIH3T3 cells, together with the BAC clone containing a transposon within the transactivation domain of RTA. Recombinant viruses in the supernatant were used to infect NIH3T3 cells. Circular BAC DNA was purified and electroporated into DH10B cells. *Chl*, chloramphenicol; *Kan*, kanamycin. (B and C) BACs containing γHV68 genome were analyzed by *Kpn*I (B) or *Eco*RI (C) digestion, and resolved on 0.8% agarose gels stained with ethidium bromide. The white arrows indicate the specific fragment shift caused by homologous recombination within the RTA locus. NR, RTA-null rescued. (D) 293T cells were transfected with γHV68 M3 reporter plasmid and BAC.NR or BAC.TTS/A. At 28 h post-transfection, luciferase activity and β-galactosidase activity were determined and M3 transcriptional activation by RTA was shown. (E) Transfection of 293T cells with the BAC.TTS/A DNA and a plasmid containing TRAF6, and qRT-PCR were carried out as in Figure 4F.

To further investigate the roles of RTA phosphorylation, we assessed the effects of the STS/A and TTS/A mutations on γHV68 lytic replication. Taking advantage of the γHV68-containing BAC with a transposon insertion that inactivates RTA (ORF50 Null) [32], a recombination-based strategy [33] was employed to generate viruses carrying wild-type RTA (Null Rescued, designated NR), the STS/A allele, or the TTS/A allele (Figure 6A). Whereas we easily obtained recombinant γHV68 containing wild-type RTA (γHV68.NR) or the TTS/A allele (γHV68.TTS/A), the STS/A variant failed to support γHV68 recombination in multiple independent experiments. This observation suggests an essential role for the phosphorylated STS sequence in γHV68 lytic replication. To confirm the integrity of viral genomic DNA, we performed restriction digestion with *Kpn*I and *Eco*RI, and analyzed with agarose gel electrophoresis. As expected, the removal of the Kanamycin cassette within RTA alleles reduced the 9-kb fragment to 7.5-kb counterpart released by *Kpn*I digestion (Figure 6B), and abolished an *Eco*RI site within the Kanamycin cassette (Figure 6C). To assess the transcriptional activity of RTA derived from BAC DNA, BAC DNA and the M3p luciferase reporter plasmid were transfected into 293T cells and RTA transcriptional activity was assessed by luciferase reporter assay. The activity of wild-type RTA to activate M3 promoter was approximately 6-fold higher than that of the TTS/A mutant (Figure 6D). Using 293T cells transfected with the γHV68 BAC containing the TTS/A allele and a plasmid expressing TRAF6, we assessed the effects of TRAF6 (that activates IKKβ) on γHV68 gene expression. In contrast to what was observed for the γHV68 BAC containing wild-type RTA (Figure 4F), exogenous TRAF6 had marginal effects on the levels of viral mRNAs transcribed from γHV68 BAC containing the TTS/A allele (Figure 6E). These findings are consistent with the observation that IKKβ failed to further promote the transcription of the TTS/A variant (Figure 5E), supporting the conclusion that the TTS residues constitute an IKKβ phosphorylation sequence by which RTA-dependent transcription is positively regulated.

**Figure 7:**
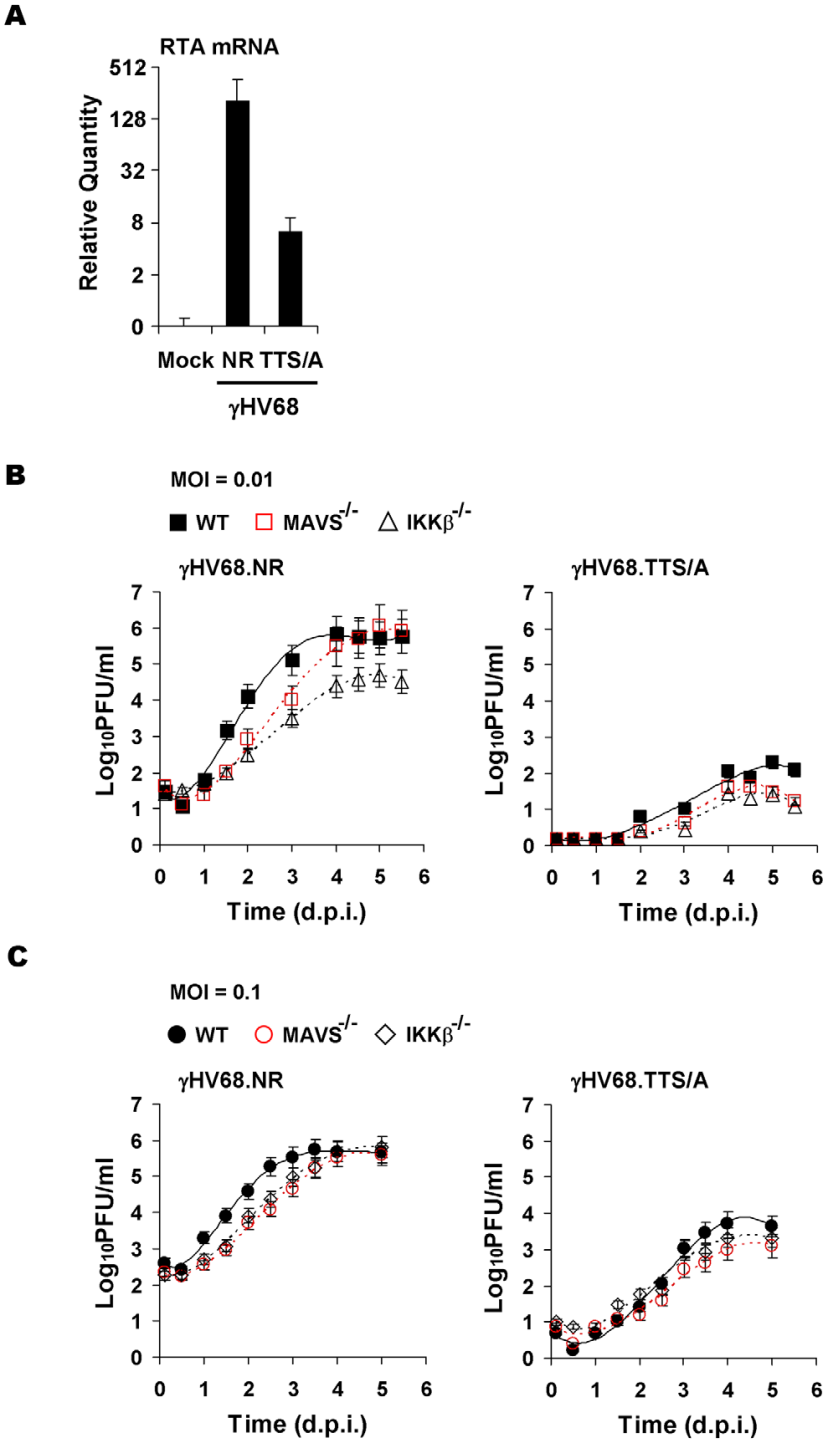
Impaired lytic replication of recombinant γHV68 carrying the TTS/A mutation. (A) Wild-type MEFs were infected with equal number of genomes, measured by qRT-PCR, of recombinant γHV68.NR (MOI = 0.1) or γHV68.TTS/A. At 30 h post-infection, the RTA mRNA levels were determined by reverse transcription and qRT-PCR. (B) Wild-type, MAVS^−/−^, and IKKβ^−/−^ MEFs were infected with recombinant γHV68.NR (left) and γHV68.TTS/A (right) (MOI = 0.01) and the multi-step growth curves were determined by a plaque assay. Data represent three independent experiments. (C) The lytic replication of recombinant γHV68.NR and γHV68.TTS/A was examined similarly as in (B) with an MOI of 0.1 (γHV68.NR).

Next, we examined whether recombinant γHV68.TTS/A recapitulates the defects of wild-type γHV68 lytic replication in MEFs deficient in MAVS and IKKβ (plaque assays and multi-step growth curves). To assess the effects of the TTS/A mutation on γHV68 transcription activation, we normalized viral genomes immediately after γHV68 *de novo* infection of MEFs by qRT-PCR. With equal number of viral genomes, γHV68.NR displayed approximately 32-fold higher of RTA mRNA than recombinant γHV68.TTS/A in MAVS^+/+^ MEFs at 30 h.p.i. (Figure 7A). This is consistent with the observation that RTA activates its own promoter to facilitate viral lytic replication (Figure 5C and 5E). Furthermore, multi-step growth curves (at an MOI of 0.01) demonstrated that γHV68.TTS/A had delayed replication kinetics and produced >3 orders of magnitude less virion progeny in MAVS^+/+^ MEFs (Figure 7B). To test whether RTA phosphorylation and the MAVS-IKKβ pathway are functionally redundant, we examined the replication kinetics of recombinant γHV68.NR and γHV68.TTS/A in wild-type, MAVS^−/−^, and IKKβ^−/−^ MEFs. Consistent with our previous observations (Figure 1C, 2C, and S3B), γHV68.NR showed delayed lytic replication in MAVS^−/−^ and IKKβ^−/−^ MEFs (Figure 7B and 7C). Remarkably, γHV68.TTS/A replicated with similar kinetics in wild-type, MAVS^−/−^, and IKKβ^−/−^ MEFs, suggesting that the MAVS-IKKβ pathway functions on RTA to promote viral lytic replication (Figure 7B and 7C). However, these replication defects of recombinant γHV68 carrying the TTS/A mutation are much more pronounced than the phenotypes of wild-type γHV68 in MAVS^−/−^ and IKKβ^−/−^ MEFs, implying that additional kinases may influence RTA transcriptional activation via phosphorylation of the TTS site. Taken together, we conclude that the TTS site of RTA is likely phosphorylated by IKKβ and is crucially important for γHV68 lytic replication.

## Discussion

Here we provide evidence that murine γHV68 hijacks the antiviral MAVS-IKKβ pathway to promote its lytic replication. The MAVS adaptor is important for host defense against invading pathogens, including various DNA and RNA viruses. For example, mice lacking MAVS were severely compromised in innate immune defense against VSV infection, leading to an elevated peak viral load and prolonged acute viral infection [34]. The antiviral effects of MAVS have been observed against the infection of a number of RNA and DNA pathogens [35], [36], [37]. To our surprise, γHV68 viral load in the lungs of MAVS^−/−^ mice was significantly lower than that in the lungs of MAVS^+/+^ mice at 10 d.p.i. The reduced viral load of γHV68 in MAVS^−/−^ mice is counter-intuitive to the presumed antiviral function of the MAVS adaptor in promoting innate immune responses. Although type I interferons in γHV68-infected mice were undetectable [38], mice deficient in type I IFN receptor had higher viral loads and succumbed to γHV68 infection [39]. We surmise that the effects of MAVS deficiency on γHV68 acute infection is likely under-estimated, providing that MAVS is critical for interferon production in response to viral infection. Thus, the viral load of γHV68 acute infection in MAVS^−/−^ mice likely represents a “neutralized” phenotype, in which reduced γHV68 lytic replication is compensated by the lack of type I interferon inhibition. Moreover, the observation that viral RTA mRNA levels correlates tightly with the MAVS mRNA levels during early γHV68 acute infection suggests that MAVS is necessary for γHV68 lytic replication (Figure S2). Although we have not formally excluded the contribution of host immune responses against γHV68 infection to the reduced viral load at 10 d.p.i. in MAVS^−/−^ mice, our experiments with γHV68 replication *ex vivo* demonstrated critical roles of the MAVS-IKKβ pathway in facilitating γHV68 lytic infection.

During early stages of viral infection, γHV68 activated IKKβ in a MAVS-dependent manner, a signaling event that is likely triggered by a variety of pathogens. The MAVS-dependent activation was supported by elevated IKKβ kinase activity and accelerated IκBα degradation, signature signaling events downstream of the MAVS adaptor. Although the up-regulation of IKKβ kianse activity appears modest, γHV68 may direct IKKβ kinase activity to efficiently modify cellular and viral components that are critical for γHV68 infection, such as RTA. Consequently, γHV68 can harness activated IKKβ without inducing NFκB activation that may be resulted from massive IKKβ activation. Indeed, it was reported that γHV68 infection does not induce NFκB activation during early infection [40], suggesting that modest IKKβ activation is beneficial for γHV68 infection and that γHV68 may uncouple NFκB activation from IKKβ activation. Interestingly, γHV68 appears to block the interferon limb of the MAVS-dependent innate immune pathway. In fact, we found that γHV68 infection failed to induce the expression of IFN-β (Figure S5A). Consistent with this observation, γHV68 RTA, similar to KSHV RTA [30], is sufficient to reduce IRF3 protein (Figure S5B), potentially abrogating the production of interferons that otherwise would potently thwart γHV68 replication. Moreover, ORF36 was reported to deregulate the phosphorylated form of IRF3 and inhibit interferon production [41]. These observations suggest that γHV68 selectively activates the MAVS-IKKβ pathway to promote viral lytic replication.

Within this report, we have identified one requisite role of the MAVS-IKKβ pathway in γHV68 lytic replication with MEFs deficient in key components of this pathway. Phenotypically, γHV68 displayed similar replication defects in MEFs deficient in MAVS, IKKβ, and IKKγ, although the replication defects in IKKβ^−/−^ and IKKγ^−/−^ MEFs were more pronounced than those in MAVS^−/−^ MEFs (Figure 1C, 2B, and 2C). This result supports the corollary that IKKβ, with the scaffold protein IKKγ, functions downstream of MAVS and likely integrates additional signaling emanating from other innate immune pathways including Toll-like receptors. It is worthy to point out that our result does not exclude the antiviral activity of the IRF-IFN pathway in γHV68 lytic replication, although deficiency of IRF3 and IRF7 or IFNAR did not appear to impact the initiation of γHV68 lytic infection as assessed by plaque assays (Figure 2B). It is possible that the IRF-IFN pathway may inhibit molecular events other than the initiation of lytic replication and reduce viral yield during γHV68 infection. Mechanistically, we identified γHV68 RTA, the master viral replication transactivator, as one of the IKKβ kinase substrates. Phosphorylation of RTA by IKKβ increases RTA transcriptional activity and consequently viral mRNA production. Indeed, γHV68 had lower levels of various mRNA transcripts that correlated with reduced lytic replication in MAVS^−/−^ MEFs (Figure 1 and 4). Conversely, exogenous TRAF6 potentiated RTA transcriptional activity and substantially increased the levels of viral mRNA transcripts (Figure 4F and 5C). Additionally, exogenously reconstituted expression of MAVS and IKKβ restored RTA phosphorylation (Figure 5B) and restored γHV68 lytic replication (Figure 1 and 2). Moreover, lytic replication of recombinant γHV68 viruses carrying mutations within the IKKβ phosphorylation sites was greatly impaired, displaying phenotypes that are more pronounced than those of wild-type γHV68 in MEFs deficient in components of the MAVS-IKKβ pathway. Conceivably, other kinases and signaling pathways may converge to modulate RTA transcriptional activation via phosphorylation within these identified IKKβ sites. For example, virus-encoded kinases, such as the functionally conserved ORF36, may amplify the phosphorylation cascade that is initiated by the MAVS-IKKβ pathway [42]. Most importantly, RTA auto-activates its own promoter and increases RTA protein that, in turn, up-regulates the expression of numerous immediate early and early genes during γHV68 infection. Thus, the 50–80% reduction in RTA transcriptional activity of the STS/A and TTS/A variants (Figure 5F) likely translates into, through the aforementioned amplification cascades, the viral yields that are less than 0.1% of the recombinant γHV68.NR (Figure 7B). Finally, it is noteworthy that deficiency in MAVS and IKKβ and mutations within RTA exhibited distinct phenotypes (such as peak viral titers of multi-step growth curves), in addition to the shared reduction of γHV68 lytic replication. These differing effects on γHV68 infection are likely due to their unique hierarchical position within the MAVS-IKKβ-RTA signaling axis. In essence, these experiments identified novel phosphorylation sites within RTA that couples γHV68 lytic replication to the antiviral IKKβ kinase. These findings collectively demonstrate that the MAVS-dependent IKKβ kinase activity is critical for RTA transcriptional activation and γHV68 lytic replication. Interestingly, Gwack et al. reported that phosphorylation of the internal serine/threonine-rich region of KSHV and γHV68 RTA inhibited RTA transcriptional activity and suppressed viral lytic replication [43]. Together with our findings, these results indicate that site-specific phosphorylation determines the transcriptional activity, and likely the promoter-specificity, of gamma-herpesvirus RTA.

Although it is well accepted that the NFκB pathway is crucial for gamma-herpesvirus latent infection [44], the roles of this pathway in gamma-herpesvirus lytic replication appear to be inconsistent. Particularly, Krug et al. reported that the recombinant γHV68 expressing the IκBα super suppressor replicated indistinguishably compared to wild type γHV68 [31]. Thus, the authors concluded that the NFκB pathway is dispensable for γHV68 lytic replication. By contrast, it was shown that RelA, the p65 subunit of an NFκB transcription dimer, inhibits γHV68 lytic replication through suppressing RTA transcription activity in 293T cells [45]. Finally, our current report indicates that the MAVS-IKKβ pathway is necessary for efficient γHV68 lytic replication. However, the seemingly paradox can be explained by the differential effects of three distinct components of the NFκB pathway on γHV68 lytic replication. Although the IκBα super suppressor is commonly employed to inhibit the activation of the NFκB transcription factors, it is important to note that no significant NFκB activation was observed during early γHV68 infection (within the first 6 hours post-infection) [40], temporal phase in which the critical roles of IKKβ was indentified by our genetic and biochemical experiments. Conceivably, the unphosphorylatable IκBα super suppressor may not impact IKKβ kinase activity. By contrast, we have focused on the IKKβ kinase and our study indicated that the ability of IKKβ to promote viral lytic replication largely stems from IKKβ kinase activity to phosphorylate RTA and increase RTA transcriptional activation. Apparently, neither IκBα, nor RelA can do so in replace of IKKβ function. On the other hand, although RelA was shown to suppress γHV68 lytic replication [45], the lack of NFκB activation during early γHV68 infection implies that γHV68 uncouples NFκB activation from IKKβ activation, which are otherwise tightly correlated. As such, γHV68 infection may selectively activate the IKKβ kinase, while sparing the inhibition by preventing NFκB activation. Therefore, a scenario that potentially accommodates all three reports is that nuclear activated RelA is necessary to inhibit γHV68 lytic replication and γHV68 is capable of preventing RelA activation in an IκBα-independent manner. Crucial to this hypothesis is the mechanisms that γHV68 has evolved to thwart NFκB activation and future experiments are necessary to address this possibility.

It was previously reported that γHV68 was impaired for latency establishment and reactivation in MyD88-deficient mice, although the lytic replication of γHV68 appeared to be normal in these mice [26]. Moreover, agonists specific for TLR7/8, which activate downstream signaling events through MyD88, induced KSHV lytic gene expression and reactivated KSHV replication from latently-infected B cells [25]. The specific roles of MAVS in lytic replication and MyD88 in latent infection are consistent with their distinct functions in innate immune responses of epithelial cells and immune cells, respectively. Given that MyD88 also activates the IKKα/β kinase complex, it is possible that IKKβ-dependent activation of RTA may contribute to γHV68 and KSHV latent infection as well. Finally, reduced lytic replication of human KSHV and cytomegalovirus has been observed under experimental conditions in which IKKβ was inhibited by Bay11, implying that human KSHV and cytomegalovirus have evolved similar molecular mechanisms to facilitate lytic replication [46], [47], [48]. Taken together, the mechanism whereby an antiviral innate immune signaling pathway is exploited to promote viral lytic replication may be applied to other herpesviruses and viral reactivation from latency. This study thus has uncovered an intricate interplay between the viral replication transactivator, RTA, and the MAVS-IKKβ pathway. To our best knowledge, this is the first example that illustrates how a virus hijacks an antiviral signaling pathway, downstream of cytosolic sensors, to initiate its lytic replication. Perhaps, co-evolution between the persistent herpesviruses and their hosts has selected viruses that exploited the inevitable innate immune activation by viral infection. Although our current study delineates the key signaling events downstream of MAVS and IKKβ, it remains unknown what viral components and cellular factors activate the MAVS-IKKβ pathway and whether these mechanisms are shared by the oncogenic KSHV and EBV to promote lytic replication or reactivation.

## Materials and Methods

### Plasmids

For protein expression in mammalian cells, all genes were cloned into pcDNA5/FRT/TO (Invitrogen) unless specified. For protein expression and purification in *E.coli*, the internal region (RTA-M, aa 335–466) and C-terminal transactivation domain (RTA-C, aa 457–583) of RTA were cloned into pGEX-4T-1 (Promega) with *Bam*HI and *Xho*I sites.

### Cells and Viruses

NIH3T3 cells, HEK293T (293T) cells and mouse embryonic fibroblasts (MEFs) were maintained in DMEM (Mediatech) with 8% newborn calf serum (NCS) or fetal bovine serum (FBS), respectively. MAVS^+/+^, MAVS^−/−^, IKKβ^−/−^, IKKγ^−/−^ and TRAF6^−/−^ MEFs were described previously [4], [34]. IKKα^−/−^ MEFs were kindly provided by Dr. Amyn A. Habib (Neurology, UT Southwestern). IFNAR^+/+^ and IFNAR^−/−^ MEFs were kindly provided by Dr. Michael Gale (Immunology, University of Washington). IRF3^+/+^IRF7^+/+^ and IRF3^−/−^IRF7^−/−^ MEFs were kindly provided by Dr. Jae Jung (Microbiology, University of Southern California). γHV68 K3/GFP was kindly provided by Dr. Philip Stevenson (Cambridge University, UK). Wild-type γHV68 and γHV68 K3/GFP were amplified in NIH3T3 cells, and VSV-GFP virus was amplified in BHK-21 cells. Viral titer was determined by a plaque assay with NIH3T3 cells.

### Mice and Infections

All animal experiments were performed in accordance to NIH guidelines, the Animal Welfare Act, and US federal law. The experimental protocol (entitled: Innate immune pathways in γHV68 infection) were approved by the Institutional Animal Care and Use Committee (IACUC). All animals were housed in a centralized research animal facility that is accredited by the Association of Assessment and Accreditation of Laboratory Animal Care International, and that is fully staffed with trained husbandry, technical, and veterinary personnel.

Wild-type (MAVS^+/+^), heterozygous (MAVS^+/−^), and knockout (MAVS^−/−^) mice were described previously [34]. Gender-matched, 6- to 8-week old littermate mice were intranasally (i.n.) inoculated with 40 plaque-forming unit (PFU) wild-type γHV68. To assess MAVS expression in the lung and spleen, BL/6 mice were intranasally infected with 1×10^5^ PFU γHV68. The lungs and spleens were harvested and homogenized in DMEM.

### Plaque Assay

Viral titer of mice tissues or cell lysates was assessed by a plaque assay on NIH3T3 monolayers. After three rounds of freezing and thawing, 10-fold serially-diluted virus supernatants were added onto NIH3T3 cells and incubated for 2 hours at 37°C. Then, DMEM containing 2% NCS and 0.75% methylcellulose (Sigma) was added after removing the supernatant. Plaques were counted at day 6 post-infection. The detection limit for this assay is 5 PFU. To assess the infectivity of γHV68 on various MEFs, a similar plaque assay was carried out with the initial cell density of 5000 cells/cm^2^. In Bay11-7082 treatment assay, 0.5µM or 1µM Bay11-7082 was added at 0.5 h before infection or 7 h post-infection. Supernatant was removed after 30 min incubation at 37°C, and cells were washed with medium and incubated for plaque formation.

### Protein Expression and Purification

Glutathione-*S*-transferase (GST) and GST fusion proteins containing the internal region and the transactivation domain of RTA were expressed with IPTG induction and purified with glutathione-sepharose as previously described [33]. Eluted proteins were re-suspended in 25% glycerol and stored at −20°C for kinase assays. To purify IKKβ and IKKβΔKD, 293T cells were transfected with pcDNA3 containing Flag-IKKβ and Flag-IKKβΔKD. At 48 h post-transfection, cells were lysed with kinase purification buffer (150 mM NaCl, 20 mM Tris.HCl pH7.4, 10% Glycerol, 0.5% Triton X-100, 0.5 mM DTT) and subject to one-step affinity purification with anti-Flag M2-conjugated agarose (Sigma). Proteins were eluted with 0.2 mg/ml Flag peptide in kinase buffer (50 mM KCl, 2 mM MgCl_2_, 2 mM MnCl_2_, 1 mM DTT, 10 mM NaF, 25 mM HEPES, pH7.5) and stored in 25% glycerol at −80°C.

### Antibodies

Commercial antibodies used in this study include: anti-Flag (Sigma), anti-GFP (Covance), anti-IKKβ (H4), anti-IκBα (C20) (Santa Cruz Biotech.), anti-actin (Abcam.). To generate antibody to γHV68 RTA, the mixture of GST fusion proteins containing the RTA-M and RTA-C was used to immunize a rabbit and polyclonal antibodies were tested for the specificity with pre-immune serum as control.

### *In Vitro* Kinase Assay

Endogenous IKKβ or exogenously expressed IKKβ and IKKβΔKD were used for *in vitro* kinase assays. The kinase reaction includes 0.5 µg GST or GST fusion proteins, 100 µCi [^32^P]γATP, and approximately 250 ng kinase in 20 µl of kinase buffer. Reaction was incubated at room temperature for 25 min and denatured proteins were analyzed by SDS-PAGE and autoradiography.

### Reverse Transcription (RT)-PCR and Quantitative Real-Time PCR (qRT-PCR) Analysis

To determine the relative levels of viral transcripts, total RNA was extracted from MEFs or mice tissues using TRIzol reagent (Invitrogen). To remove genomic DNA, total RNA was treated with RNase-free DNase I (New England Biolab) at 37°C for 1 hour. After heat inactivation, total RNA was re-purified with TRIzol reagent. cDNA was prepared with 1.5 µg total RNA and reverse transcriptase (Invitrogen). RNA was then removed by incubation with RNase H (Epicentre). Abundance of viral transcripts was assessed by qRT-PCR. Mouse β-actin was used as an internal control. Primers used in this study were summarized in *Table S1*.

### Limiting-Dilution *Ex Vivo* Reactivation Analyses

Bulk splenocytes were re-suspended in DMEM, and plated onto primary MEF monolayers in 96-well plates in 2-fold serial dilutions (from 10^5^ to 48 cells/well) as previously described [49]. Twelve wells were plated every dilution. Reactivation percentage was scored for cytopathic effects (CPE) positive wells on day 6. In order to measure preformed infectious virus, disrupted cells were plated onto primary MEF monolayers. This procedure destroys over 99% of the cells, but has minimal effect on preformed infectious virus, thus allowing distinction between reactivation from latency and persistent infection.

### Limiting-Dilution Nested PCR (LDPCR) Detection of γHV68 Genome-positive Cells

The frequency of splenocytes harboring wild-type γHV68 genome was assessed by a single-copy-sensitive nested PCR analysis of serial dilutions of splenocytes as previously described [49]. Briefly, mice spleens were homogenized and re-suspended in isotonic buffer and subjected to 3-fold serial dilutions (from 10^4^ to 41 cells/well) in a background of uninfected RAW 264.7 cells, with a total of 10^4^ cells per well. Twelve replicates were plated for each cell dilution. After being plated, cells were subjected to lysis by proteinase K at 56°C for 8 hours. After inactivating the enzyme for 30 minutes at 85°C, samples were subjected to nested PCR using primers specific for γHV68 ORF72. Positive controls of 10, 1, and 0.1 copies of viral DNA and negative controls of uninfected RAW 264.7 cells alone were included on each plate. Reaction products were separated using 2.5% UltraPure agarose (Invitrogen) gels and visualized by ethidium bromide staining.

### Statistical Analyses

Reactivation and LDPCR results were analyzed using GraphPad Prism software (GraphPad Software, San Diego, CA). The frequencies of genome-positive cells were statistically analyzed using the paired Student's *t*-test. The frequencies of viral genome-positive cells were determined from a nonlinear regression analysis of sigmoidal dose-response best-fit curve data. Based on a Poisson distribution, the frequency at which at least one event is present in a given population occurs at the point at which the regression analysis line intersects 63.2%. Pooled data of at least three independent experiments were used to calculate *P* values with the two-tailed, unpaired Student's *t*-test.

### Luciferase Reporter Assay

293T cells (2×10^5^ cells/well) were seeded in 24-well plates 16 hours before transfection. A total of 377 ng of plasmid DNA per well was co-transfected by the calcium phosphate method (Clontech). The plasmid cocktail includes 75 ng of luciferase plasmid (RTAp_luc, ORF57p_Luc or M3p_luc), 200 ng of pCMV-β-galactosidase plasmid, 2 ng of pcDNA5_RTA and 100 ng of pcDNA5 containing TRAF6, IKKβ or IKKβΔKD. At 21 hours post-transfection, whole cell lysates were used to measure the firefly luciferase activity and β-galactosidase activity.

### Generating Recombinant γHV68

The bacterial artificial chromosome (BAC) system was used to generate recombinant γHV68 similarly to what was described previously [33]. Briefly, wild-type RTA or the STS/A and TTS/A alleles were PCR amplified with overlapping PCR primers. Purified PCR products, along with the BAC clone 5.15 [32] containing a transposon within the transactivation domain of RTA (between nucleotide 69269 and 69270, according to accession number U97553), were transfected into NIH3T3 cells with Lipofectamine 2000 (Invitrogen). Virus in the supernatant was further amplified with NIH3T3 cells. To isolate circular BAC DNA, NIH3T3 cells were infected with recombinant γHV68 and DNA was extracted according to Hirt's protocol [50] and electroporated into ElectroMAX DH10B cells (Invitrogen). BAC DNA containing γHV68 genome was digested with *Eco*RI and *Kpn*I to rule out chromosome rearrangement. Meanwhile, the RTA alleles were amplified by PCR and sequenced to confirm desired mutations. Selected clones were transfected into NIH3T3 cells and recombinant γHV68 was amplified for subsequent experiments.

### NCBI Entrez Gene ID List

RIG-I, 230073; MDA-5, 71586; MAVS, 228607; TBK-1, 56480; IKKε, 56489; IRF3, 54131; IRF7, 54123; c-Jun, 16476; ATF-2, 11909; IFNβ, 15977; IFNAR, 15975; TRAF3, 22031; TRAF6, 22034; IKKγ, 16151; IKKα, 12675; IKKβ, 16150; IκBα, 18035; NFκB1, 18033; RelA, 19697; MyD88, 35956; TLR7, 170743; TLR8, 170744.

## Supporting Information

Figure S1 Normal γHV68 Latent Infection in MAVS^−/−^ Mice. Six- to 8-week old mice were intranasally infected with 40 PFU wild-type γHV68. Limiting dilution assays were carried out with splenocytes of MAVS^+/+^ mice (filled square and circle) or MAVS^−/−^ mice (open square and circle). Viral genome frequency (A), preformed infection and reactivation at day 16 (B) and day 45 (C) post-infection were measured as described in *Materials and Methods*. The data were compiled from two independent experiments with four mice per group per experiment, and are presented as the mean ± standard error of the mean (SEM).
(0.28 MB TIF)

Figure S2 Correlation Between γHV68 RTA mRNA Levels and MAVS mRNA Levels. (A and B) BL/6 mice were intranasally infected with 1×10^5^ PFU γHV68, and the mRNA levels of MAVS (A) and viral RTA (B) in the lungs and spleens were determined by reverse transcription and qRT-PCR using comparative C_T_ method. (C) Correlation between the viral RTA mRNA levels (B) and the MAVS mRNA levels (A) in the lungs (at 2.5 [filled circle] and 5 [open square] d.p.i.) (left) and spleens (at 5 d.p.i. [filled triangle]) (right). Dashed lines represent the linear regression between viral RTA mRNA levels and MAVS mRNA levels.
(0.23 MB TIF)

Figure S3 Delayed Lytic Replication of γHV68 in MAVS^−/−^ MEFs. MAVS^+/+^ (filled triangle) and MAVS^−/−^ (open triangle) MEFs were infected with γHV68 K3/GFP virus at the multiplicity of infection (MOI) of 0.1. (A) γHV68 infected cells were photographed at day 3 and day 5 post-infection (d.p.i.). (B) Multi-step growth curve of γHV68 was determined by a plaque assay. Results represent the mean ± SEM of three independent experiments.
(0.57 MB TIF)

Figure S4 Reduced Initiation of γHV68 Lytic Replication in MAVS^−/−^ MEFs. MAVS^+/+^ (filled circle) and MAVS^−/−^ (open circle) MEFs were infected with γHV68 K3/GFP. (A) Methylcellulose was added to block viral transmission through the supernatant at time points as indicated. Plaques formed by γHV68 were counted at day 6 post-infection. The results are expressed as the mean ± SEM of three independent samples. (B to D) Methylcellulose was added at 2 hours post-infection. (B) Plaques were counted after infection of indicated dose of γHV68. The results are expressed as the mean ± SEM of three independent samples. (C) Plaques were photographed by fluorescent microscopy. Results represent four independent wells of both cell lines. (D) Plaque dimensions were measured on MAVS^+/+^ (n = 86) and MAVS^−/−^ MEFs (n = 81). Each solid or open circle represents a plaque.
(1.12 MB TIF)

Figure S5 γHV68 does not Induce IFNβ Gene Expression in MEFs. (A) MAVS^+/+^ and MAVS^−/−^ MEFs were infected with γHV68 (MOI = 5) or Sendai virus (150 HA Units) separately. IFNβ mRNA levels were determined by reverse transcription and quantitative real-time PCR. (B) 293T cells were transfected with plasmids carrying IRF3-GFP and γHV68 RTA. IRF3 protein levels after 48 hours were monitored by GFP fluorescence microscopy.
(0.67 MB TIF)

Figure S6 IKKβ, but not IKKα, Phosphorylates GST-RTA-C. GST, GST-RTA-M, and GST-RTA-C were incubated with [^32^P]γATP and IKKα or IKKβ, and analyzed by coomassie staining or autoradiography. IKKα and IKKβ were analyzed by immunoblot with anti-Flag antibody.
(0.30 MB TIF)

Figure S7 TRAF6, IKKβ or IKKβΔKD does not Activate the Promoters of γHV68 Lytic Genes. (A) 293T cells were transfected with reporter plasmids and 100 ng plasmids containing TRAF6, IKKβ or IKKβΔKD. Luciferase activity was normalized against β-galactosidase activity. Data represent the mean ± SEM of at least five independent experiments. (B and C) 293T cells were transfected with reporter plasmids, RTA (2 ng) and IKKβ (100 ng) or IKKβΔKD (200, 500 and 1000 ng). (B) Whole cell lysates (WCLs) were analyzed by immunoblot with anti-Flag antibody for IKKβ and IKKβΔKD expression. The relative molar ratio between IKKβ and IKKβΔKD was calculated by normalizing immunoblot intensity to the β-galactosidase activity (β-Gal) and their molecular weights. (C) Luciferase activity normalized against β-galactosidase activity was shown. Vec, empty vector.
(0.38 MB TIF)

Figure S8 Identification of IKKβ Phosphorylation Sites within γHV68 RTA. GST and a panel of GST-RTA fusion proteins with serial truncations (top) or site-specific mutations (bottom) were analyzed by *in vitro* kinase assays with IKKβ. Relative intensity of phosphorylated GST-RTA fusion protein was quantified in reference to GST-RTA-C. ND, not done.
(0.18 MB TIF)

Table S1 Primer List. All primers used in this study were synthesized by Invitrogen or Sigma. PCR primers were designed by MacVector 9.0 (Accelrys Inc.), and qRT-PCR (quantitative Real-Time PCR) primers were designed by Primer Express 3.0 (Applied Biosystems).
(0.23 MB TIF)
