# Supplementary figures and images for: Murine Gamma-Herpesvirus 68 Hijacks MAVS and IKKβ to Initiate Lytic Replication
Source: PLoS Pathog. 2010 Jul 29;6(7):e1001001. doi: 10.1371/journal.ppat.1001001 (PMC2912392; doi:10.1371/journal.ppat.1001001)

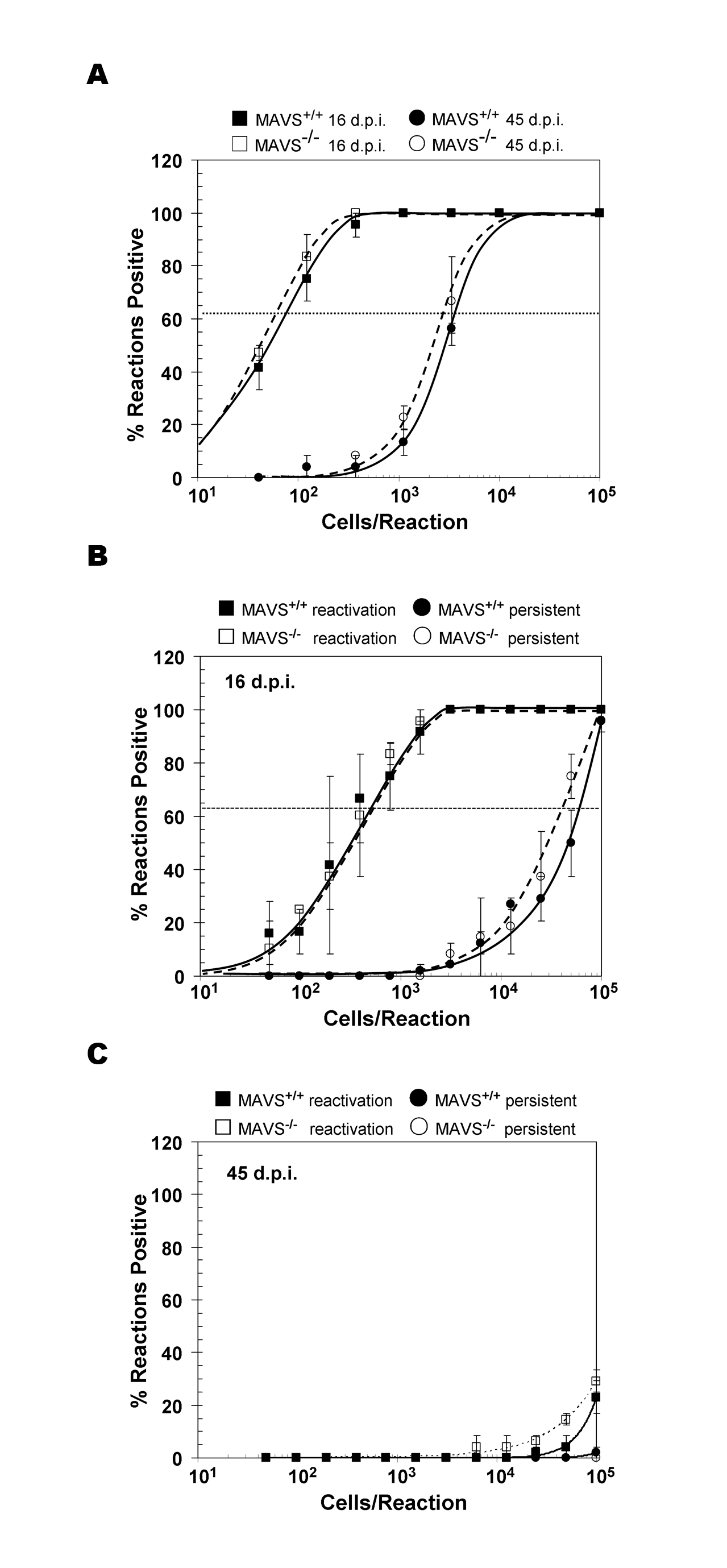

Supplement: Figure S1 — Normal γHV68 Latent Infection in MAVS−/− Mice. Six- to 8-week old mice were intranasally infected with 40 PFU wild-type γHV68. Limiting dilution assays were carried out with splenocytes of MAVS+/+ mice (filled square and circle) or MAVS−/− mice (open square and circle). Viral genome frequency (A), preformed infection and reactivation at day 16 (B) and day 45 (C) post-infection were measured as described in Materials and Methods . The data were compiled from two independent experiments with four mice per group per experiment, and are presented as the mean ± standard error of the mean (SEM). (0.28 MB TIF) [file ppat.1001001.s001.tif]

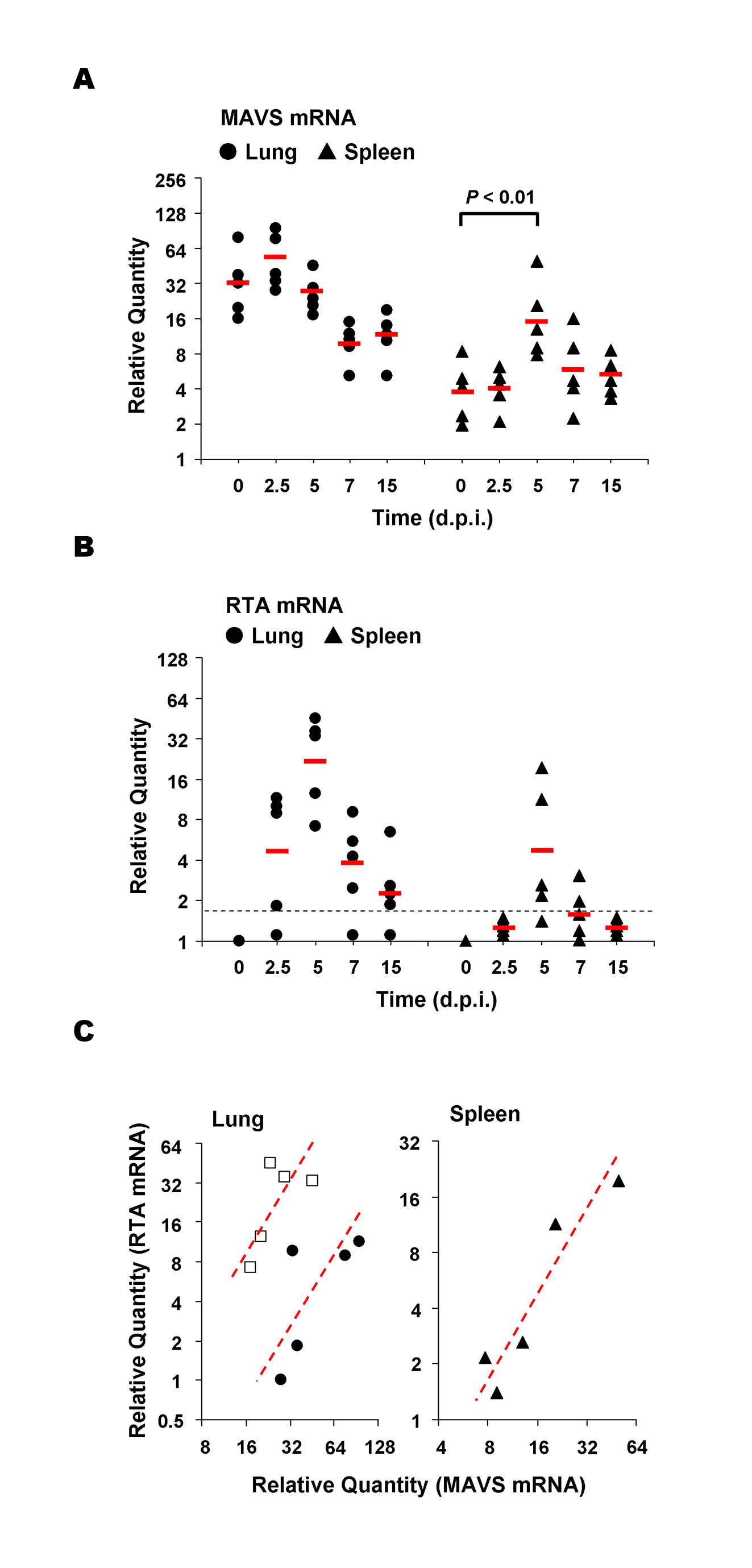

Supplement: Figure S2 — Correlation Between γHV68 RTA mRNA Levels and MAVS mRNA Levels. (A and B) BL/6 mice were intranasally infected with 1×10^5^ PFU γHV68, and the mRNA levels of MAVS (A) and viral RTA (B) in the lungs and spleens were determined by reverse transcription and qRT-PCR using comparative CT method. (C) Correlation between the viral RTA mRNA levels (B) and the MAVS mRNA levels (A) in the lungs (at 2.5 [filled circle] and 5 [open square] d.p.i.) (left) and spleens (at 5 d.p.i. [filled triangle]) (right). Dashed lines represent the linear regression between viral RTA mRNA levels and MAVS mRNA levels. (0.23 MB TIF) [file ppat.1001001.s002.tif]

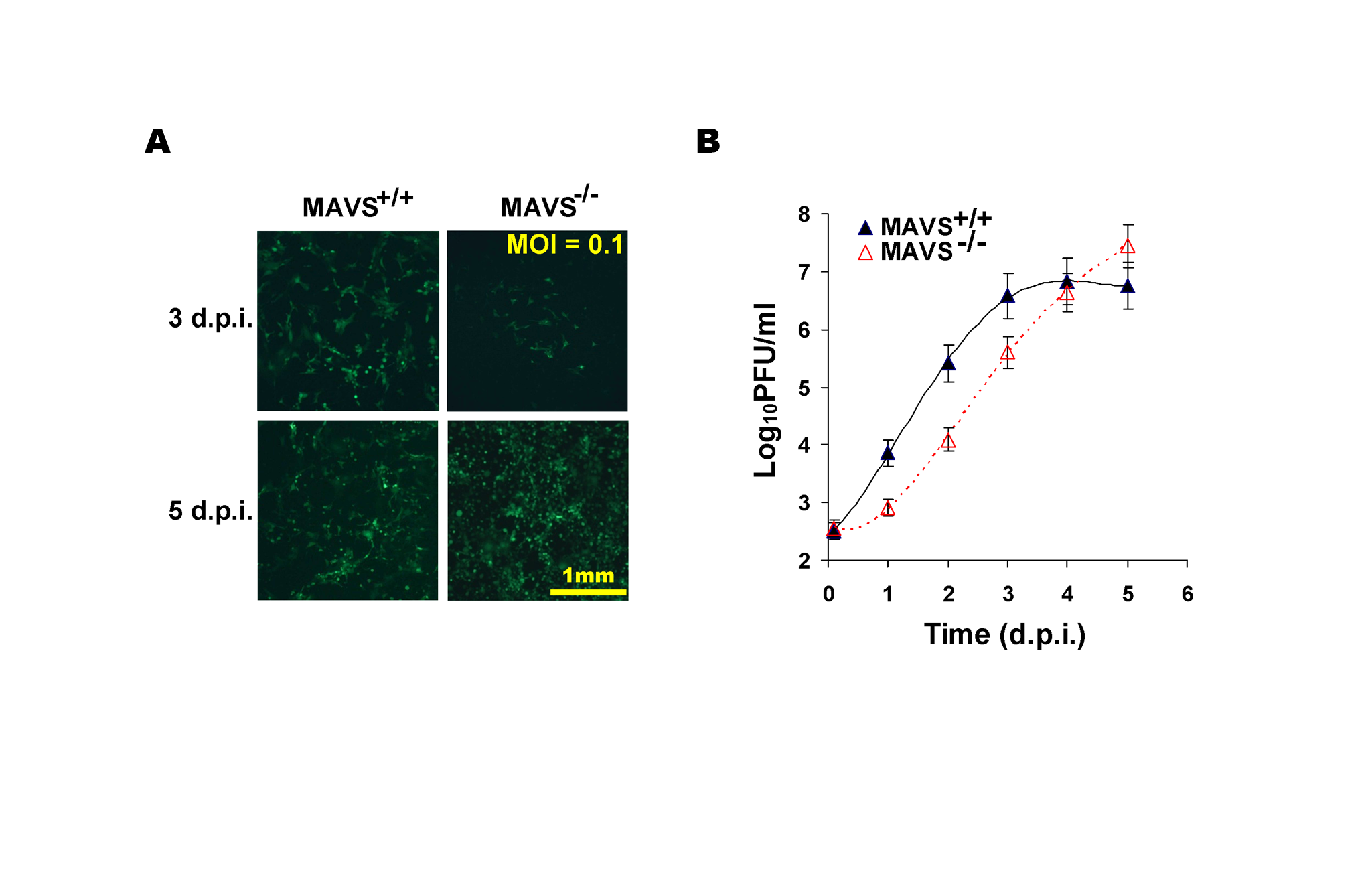

Supplement: Figure S3 — Delayed Lytic Replication of γHV68 in MAVS−/− MEFs. MAVS+/+ (filled triangle) and MAVS−/− (open triangle) MEFs were infected with γHV68 K3/GFP virus at the multiplicity of infection (MOI) of 0.1. (A) γHV68 infected cells were photographed at day 3 and day 5 post-infection (d.p.i.). (B) Multi-step growth curve of γHV68 was determined by a plaque assay. Results represent the mean ± SEM of three independent experiments. (0.57 MB TIF) [file ppat.1001001.s003.tif]

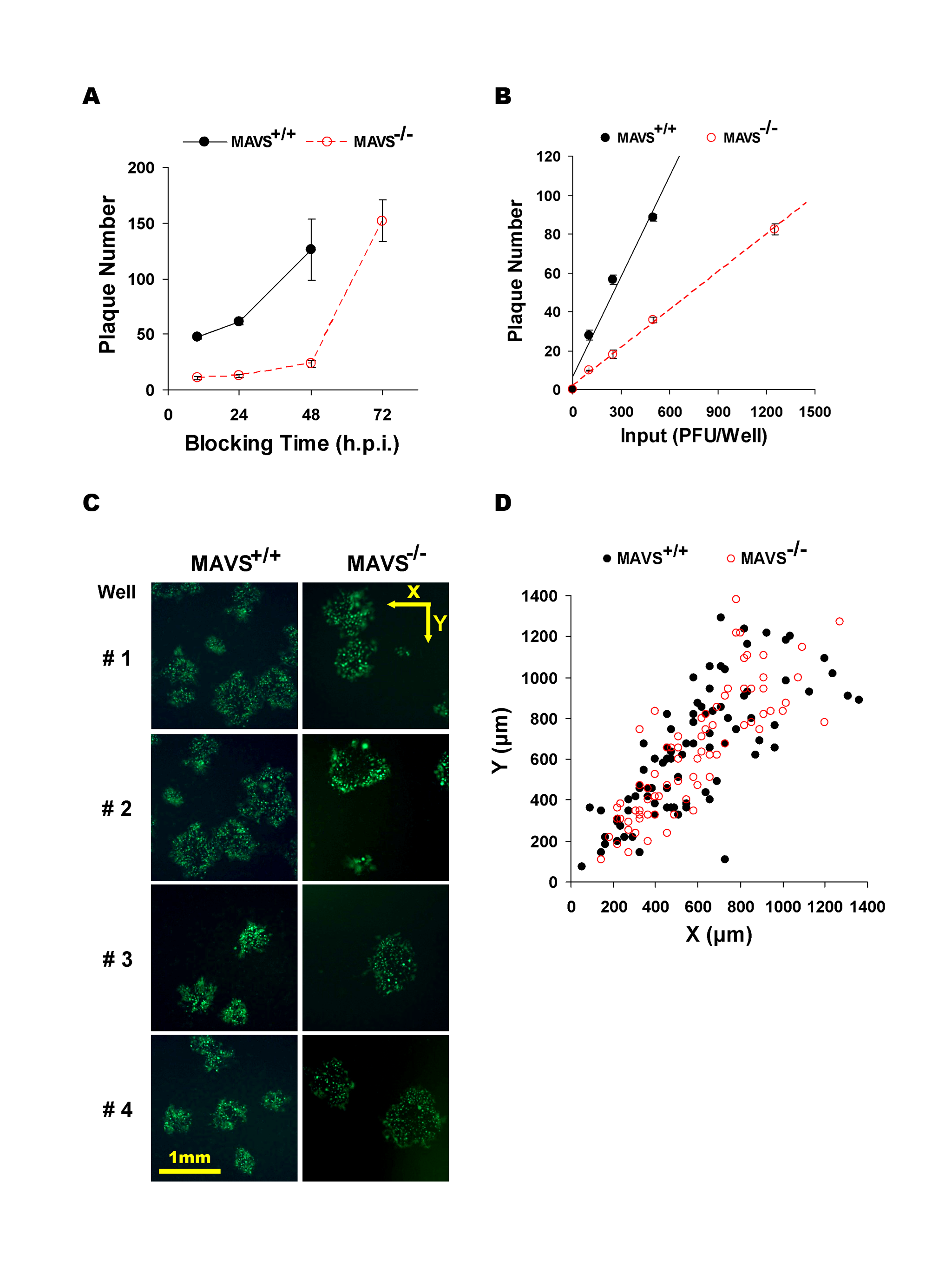

Supplement: Figure S4 — Reduced Initiation of γHV68 Lytic Replication in MAVS−/− MEFs. MAVS+/+ (filled circle) and MAVS−/− (open circle) MEFs were infected with γHV68 K3/GFP. (A) Methylcellulose was added to block viral transmission through the supernatant at time points as indicated. Plaques formed by γHV68 were counted at day 6 post-infection. The results are expressed as the mean ± SEM of three independent samples. (B to D) Methylcellulose was added at 2 hours post-infection. (B) Plaques were counted after infection of indicated dose of γHV68. The results are expressed as the mean ± SEM of three independent samples. (C) Plaques were photographed by fluorescent microscopy. Results represent four independent wells of both cell lines. (D) Plaque dimensions were measured on MAVS+/+ (n = 86) and MAVS−/− MEFs (n = 81). Each solid or open circle represents a plaque. (1.12 MB TIF) [file ppat.1001001.s004.tif]

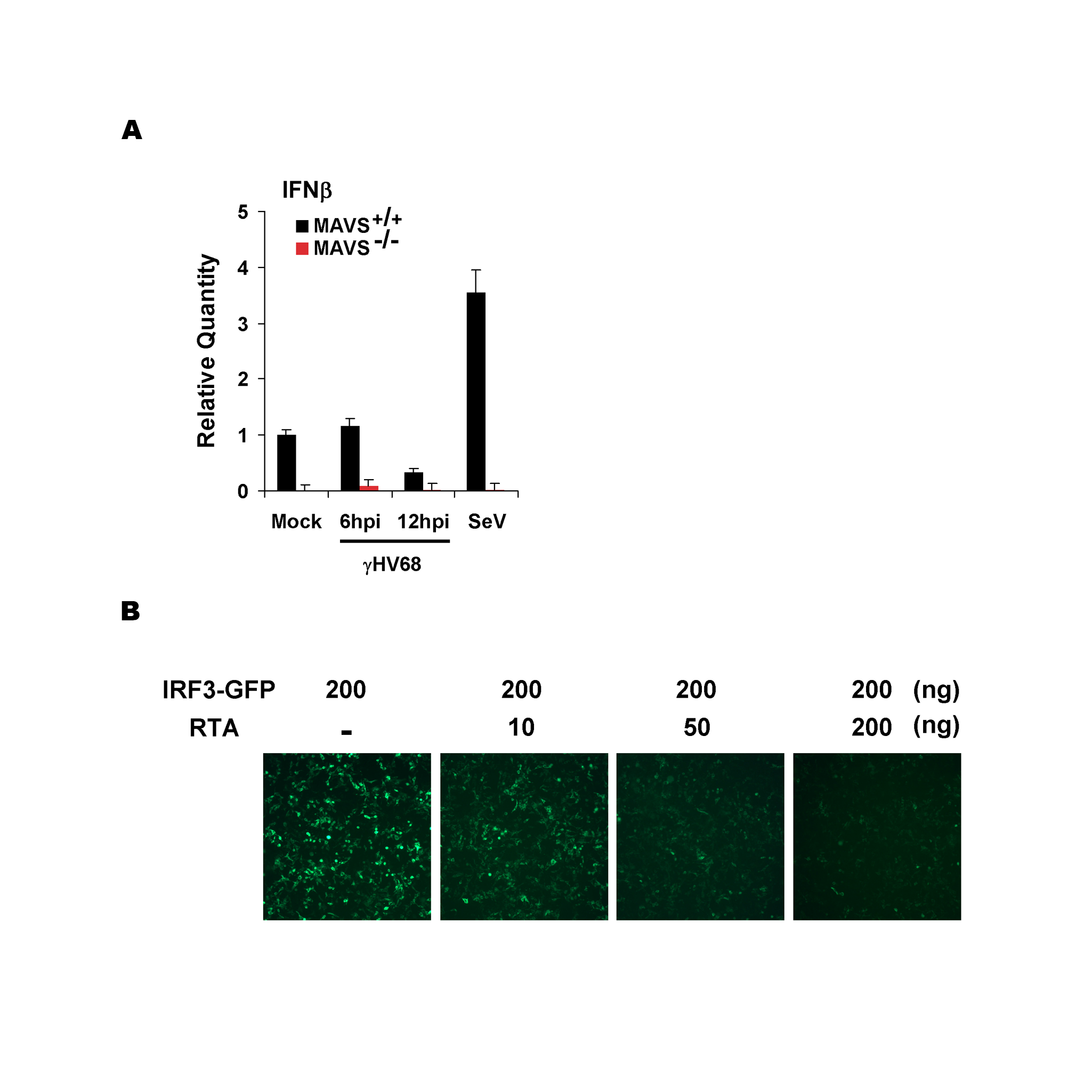

Supplement: Figure S5 — γHV68 does not Induce IFNβ Gene Expression in MEFs. (A) MAVS+/+ and MAVS−/− MEFs were infected with γHV68 (MOI = 5) or Sendai virus (150 HA Units) separately. IFNβ mRNA levels were determined by reverse transcription and quantitative real-time PCR. (B) 293T cells were transfected with plasmids carrying IRF3-GFP and γHV68 RTA. IRF3 protein levels after 48 hours were monitored by GFP fluorescence microscopy. (0.67 MB TIF) [file ppat.1001001.s005.tif]

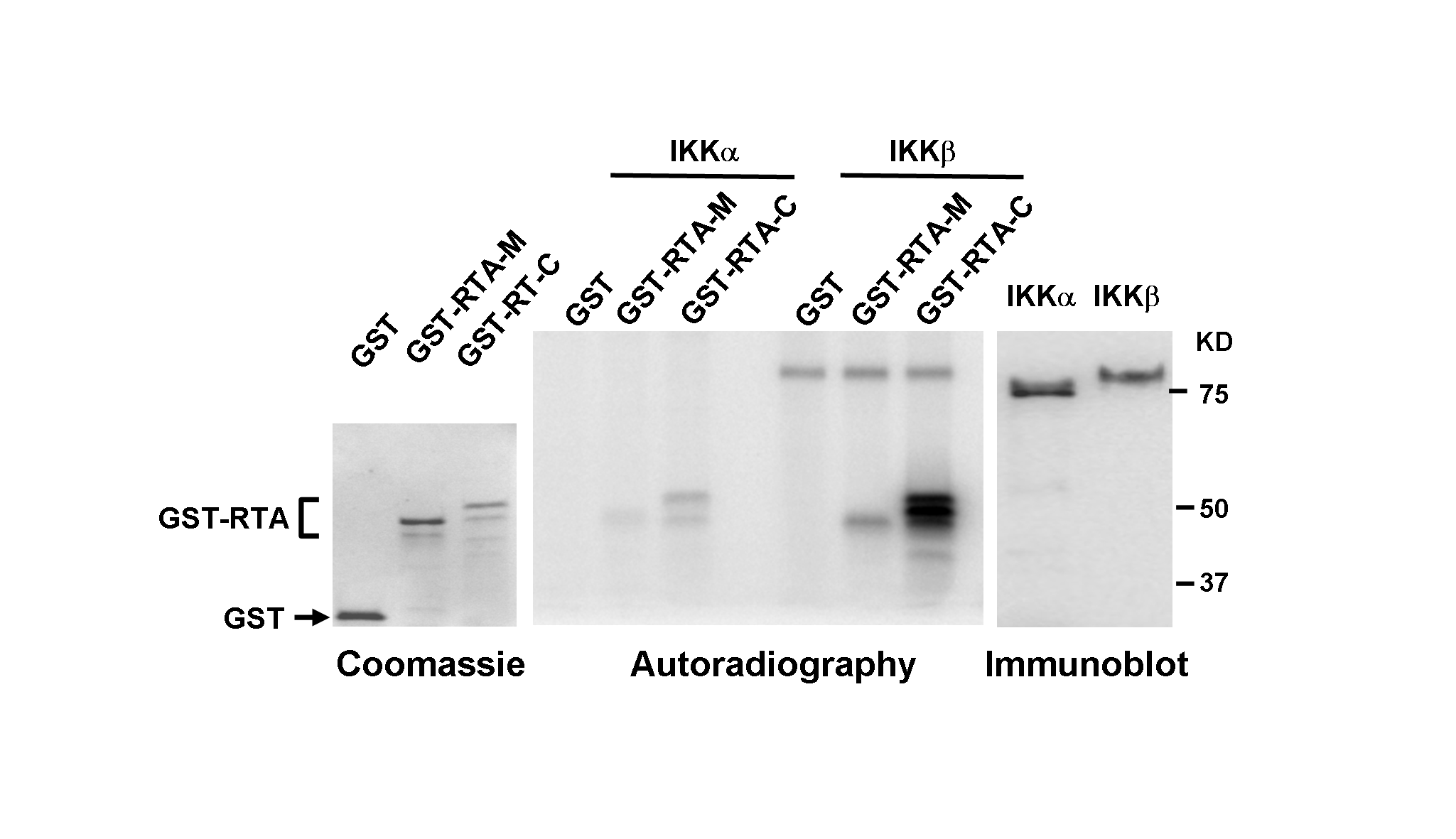

Supplement: Figure S6 — IKKβ, but not IKKα, Phosphorylates GST-RTA-C. GST, GST-RTA-M, and GST-RTA-C were incubated with [32P]γATP and IKKα or IKKβ, and analyzed by coomassie staining or autoradiography. IKKα and IKKβ were analyzed by immunoblot with anti-Flag antibody. (0.30 MB TIF) [file ppat.1001001.s006.tif]

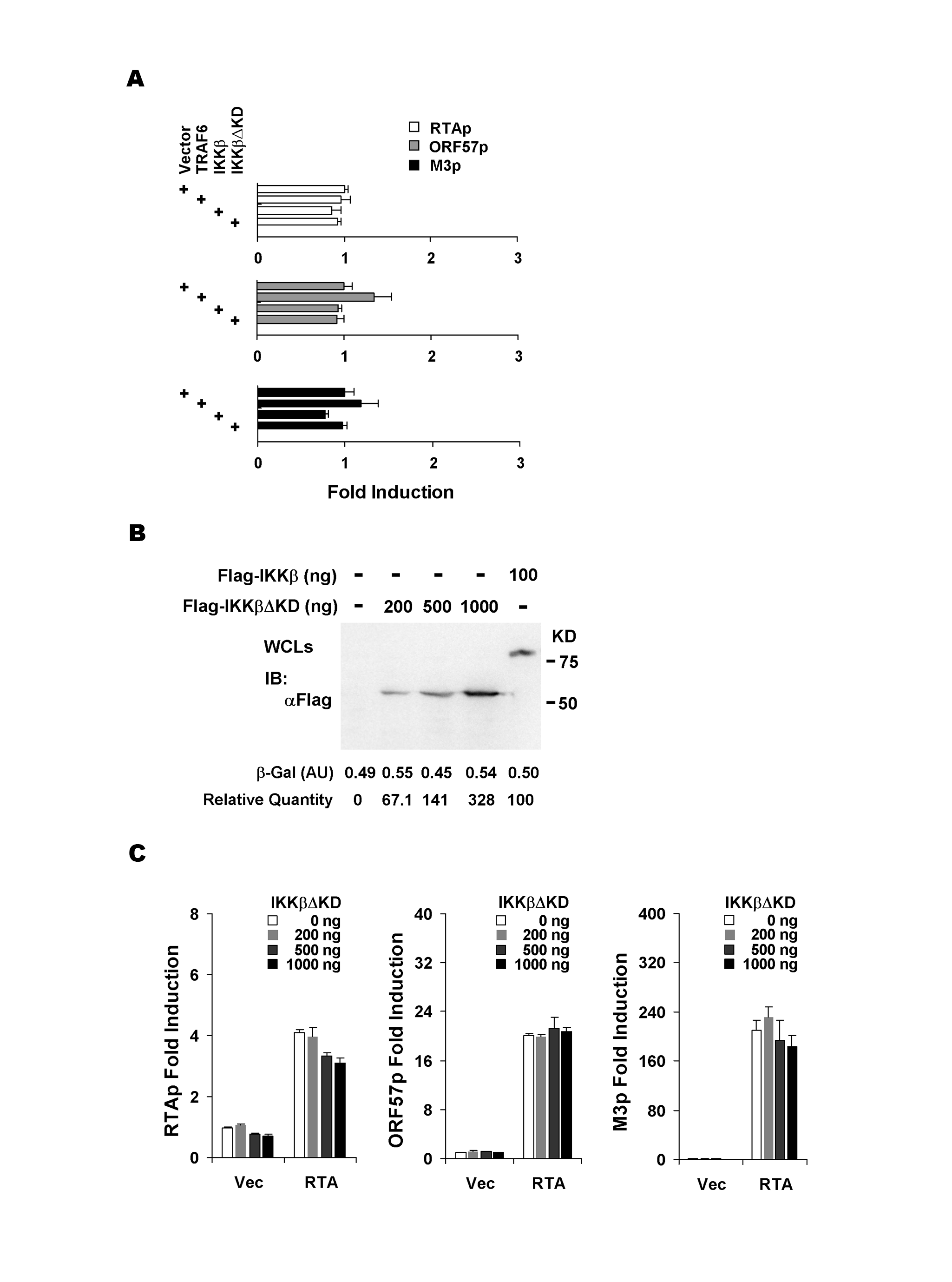

Supplement: Figure S7 — TRAF6, IKKβ or IKKβΔKD does not Activate the Promoters of γHV68 Lytic Genes. (A) 293T cells were transfected with reporter plasmids and 100 ng plasmids containing TRAF6, IKKβ or IKKβΔKD. Luciferase activity was normalized against β-galactosidase activity. Data represent the mean ± SEM of at least five independent experiments. (B and C) 293T cells were transfected with reporter plasmids, RTA (2 ng) and IKKβ (100 ng) or IKKβΔKD (200, 500 and 1000 ng). (B) Whole cell lysates (WCLs) were analyzed by immunoblot with anti-Flag antibody for IKKβ and IKKβΔKD expression. The relative molar ratio between IKKβ and IKKβΔKD was calculated by normalizing immunoblot intensity to the β-galactosidase activity (β-Gal) and their molecular weights. (C) Luciferase activity normalized against β-galactosidase activity was shown. Vec, empty vector. (0.38 MB TIF) [file ppat.1001001.s007.tif]

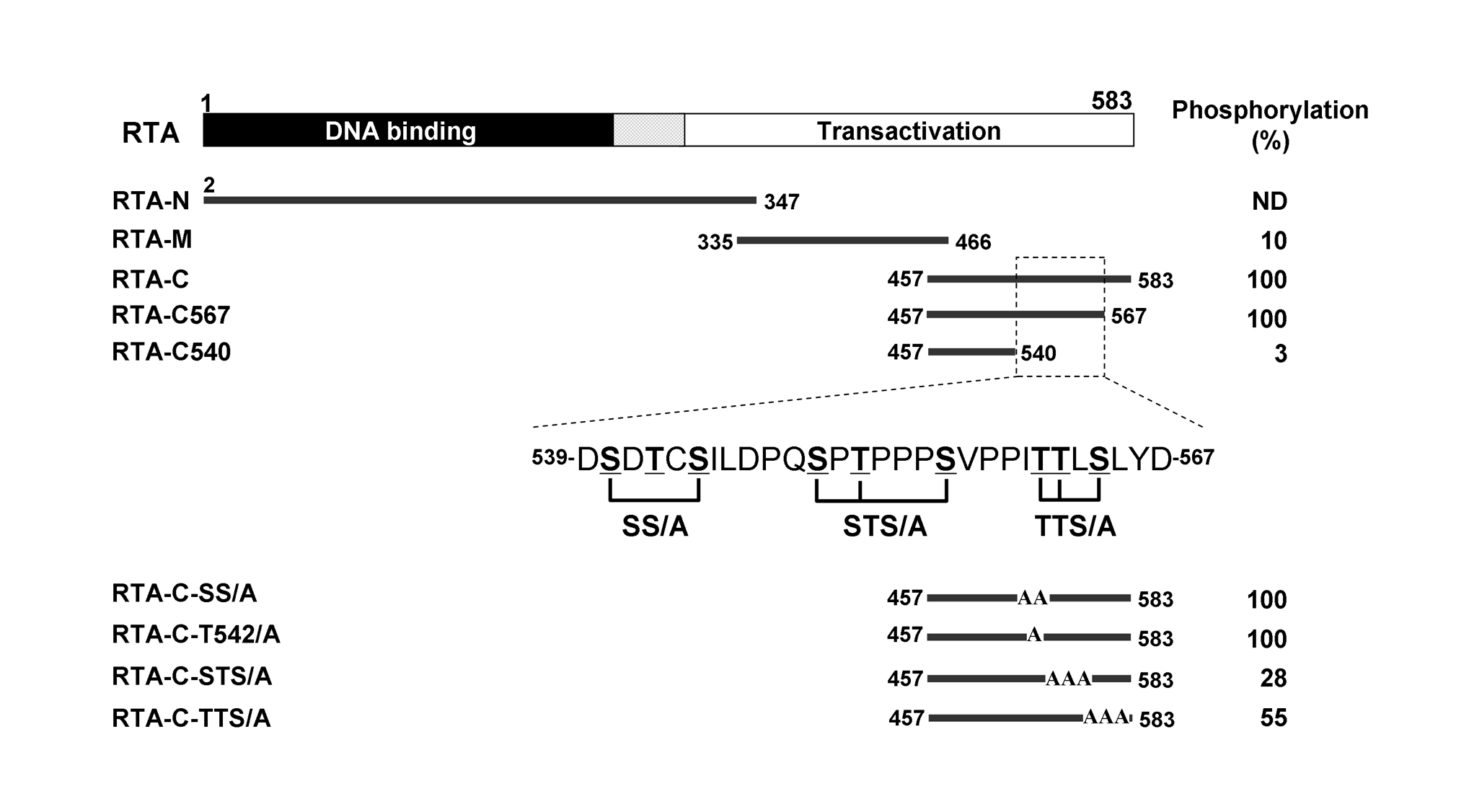

Supplement: Figure S8 — Identification of IKKβ Phosphorylation Sites within γHV68 RTA. GST and a panel of GST-RTA fusion proteins with serial truncations (top) or site-specific mutations (bottom) were analyzed by in vitro kinase assays with IKKβ. Relative intensity of phosphorylated GST-RTA fusion protein was quantified in reference to GST-RTA-C. ND, not done. (0.18 MB TIF) [file ppat.1001001.s008.tif]

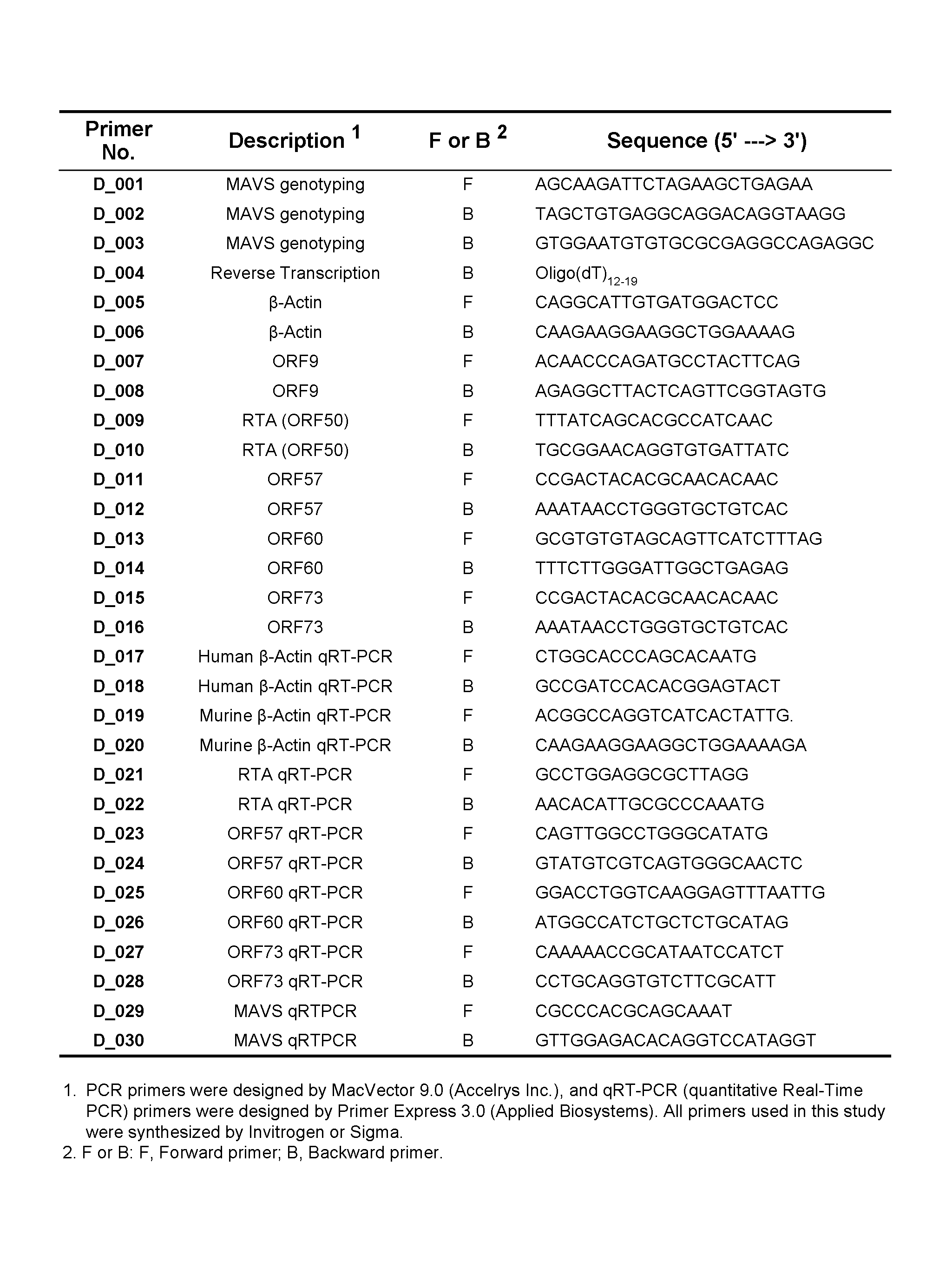

Supplement: Table S1 — Primer List. All primers used in this study were synthesized by Invitrogen or Sigma. PCR primers were designed by MacVector 9.0 (Accelrys Inc.), and qRT-PCR (quantitative Real-Time PCR) primers were designed by Primer Express 3.0 (Applied Biosystems). (0.23 MB TIF) [file ppat.1001001.s009.tif]
